# Spatial transcriptomic analysis across histological subtypes reveals molecular heterogeneity and prognostic markers in early‐stage lung adenocarcinoma

**DOI:** 10.1002/ctm2.70439

**Published:** 2025-08-22

**Authors:** Hua Geng, Wenhao Zhou, Haitao Luo, Jiaqian Wang, Shixiong Li, Congcong Song, Yujie Zhao, Meilin Xu

**Affiliations:** ^1^ Department of Pathology Tianjin Chest Hospital Tianjin China; ^2^ Kindstar Global Precision Medicine Institute Shenzhen China; ^3^ Shenzhen Engineering Center for Translational Medicine of Precision Cancer Immunodiagnosis and Therapy YuceBio Technology Co., Ltd. Shenzhen China

**Keywords:** digital spatial transcriptomic profiling, epithelial compartments, histologic subtypes, lung adenocarcinoma, macrophage compartments

## Abstract

**Background:**

The progression and prognosis of early‐stage lung adenocarcinoma are closely associated with histologic subtypes, yet the presence of mixed histologic patterns often complicates prognostic assessment. Currently, the correlation between molecular and histologic features remains poorly understood.

**Methods:**

Formalin‐fixed paraffin‐embedded (FFPE) samples were collected from patients with primary early‐stage lung adenocarcinoma, encompassing three histologic subtypes: well‐differentiated, moderately differentiated, and poorly differentiated. The GeoMx Digital Spatial Profiler platform was utilized to obtain spatial transcriptomic profiling. Regions of interest were carefully selected and further subdivided into three categories of areas of interest, specifically epithelial cell‐enriched regions, macrophage‐enriched regions, and other regions. Multiplex immunofluorescence (mIF) assays were employed to validate the obtained results.

**Results:**

Distinct molecular characteristics were identified in tumor epithelial‐ and macrophage‐enriched compartments spanning well‐differentiated to poorly differentiated tumors. In poorly differentiated tumors, we observed enrichment of pathways related to humoral immune response, complement activation regulation, and extracellular matrix receptor interaction pathways, all of which are significantly associated with poorer prognosis. We integrated these pathways to develop a composite molecular signature that strongly correlate with adverse prognosis.

**Conclusions:**

Our results provide new insights into the link between molecular and histologic subtypes in mixed‐type lung adenocarcinomas. Specifically, the identified molecular signatures offer potential biomarkers for predicting disease progression and prognosis, thus facilitating more precise and personalized therapeutic approaches.

**Key points:**

- Poorly differentiated components in mixed‐type early‐stage lung adenocarcinoma (LUAD) are characterized by enrichment of humoral immune response, complementactivation regulation, and extracellular matrix receptor interaction pathways, which areassociated with worse prognosis.
- A composite molecular signature integrating the keypathways strongly correlates with adverse clinical outcomes, serving as apotential prognostic biomarker.
- Digital spatial transcriptomics reveals spatially resolvedmolecular heterogeneity in tumor epithelial‐ and macrophage‐enrichedcompartments, bridging the gap between histologic subtypes and molecularmechanisms in LUAD.

## Graphical abstract

We observed enrichment of humoural immune response, complement activation regulation, and ECM‒receptor interaction pathways in poorly differentiated tumours, which were significantly associated with a poorer prognosis.

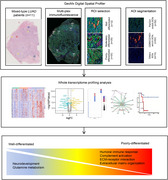

## 1 INTRODUCTION

Over the past decade, significant progress has been achieved in understanding lung cancer biology, leading to notable improvements in patient outcomes. Nevertheless, despite these advancements, lung cancer remains the leading cause of cancer‐related mortality.^1^ Lung adenocarcinoma (LUAD), a prevalent pathological subtype, exhibits remarkable histologic heterogeneity, alongside cellular and molecular diversities. This heterogeneity is further complicated by the coexistence of multiple histologic subtypes within a single tumour.^2^, ^3^ Specifically, LUAD can be categorised based on its histologic patterns into minimally invasive adenocarcinoma, invasive nonmucinous adenocarcinoma and invasive mucinous adenocarcinoma. Among these, invasive nonmucinous adenocarcinoma comprises various histologic subtypes such as lepidic, acinar, papillary, micropapillary, solid adenocarcinoma, cribriform and complex glandular adenocarcinoma patterns. These subtypes are further classified into three grades based on their degree of differentiation: well differentiated (grade 1), primarily comprising the lepidic pattern; moderately differentiated (grade 2), characterised mainly by acinar or papillary patterns; and poorly differentiated (grade 3), defined by the presence of high‐grade histological patterns (micropapillary, solid, cribriform and complex glandular patterns) accounting for more than 20% of the tumour.^4^ The presence of such a diverse range of histologic subtypes and grades within LUAD underscores the complexity of the disease and poses significant challenges in its diagnosis, treatment and management. Tumours displaying a lepidic pattern tend to have a better prognosis than those dominated by acinar or papillary adenocarcinomas. Conversely, micropapillary and solid patterns are associated with poor outcomes.^5^, ^6^, ^7^, ^8^ The complexity of LUAD, with patients often exhibiting multiple histological subtypes, challenges pathologist's reproducibility.^9^, ^10^ A detailed understanding of the molecular distinctions between these subtypes is crucial for comprehending their characteristics and enabling precise clinical treatment.

Genomic analysis of tumour compartments reveals that well‐differentiated tumours exhibit distinct mutational and oncogenic pathway alterations in micropapillary and solid tumours.^11^ Previous studies have reported epigenetic and transcriptional changes associated with the transition to aggressive patterns.^12^ Meanwhile, there is evidence that genetic alterations (e.g., chromosomal arm aberrations and loss of heterozygosity) are linked to aggressive morphological patterns.^13^ Immunological analyses link poor differentiation to immune cell infiltration and Programmed cell death 1 ligand 1 (PD‐L1) expression.^14^ Spatial omics data revealed that CD74 downregulation in malignant cells correlates with invasive progression, while macrophage‐enriched regions exhibit active MIF‒CD74 interactions in preinvasive components, suggesting a potential role for immune‒malignant crosstalk in tumour progression.^15^ However, the role of macrophages in shaping histological subtypes remains unclear. In previous studies, genomics and transcriptomics techniques have been extensively applied to lung cancer research, providing us with a wealth of gene expression information that has greatly facilitated our understanding of the molecular mechanisms underlying lung cancer.^16^, ^17^, ^18^ However, these techniques often overlook the spatial heterogeneity within tumours, that is, the potential differences in gene expression among different regions or cell types. This limitation hampers our comprehensive understanding of the molecular differences between different histologic subtypes of LUAD and how these differences influence tumour biology and patient outcomes.

To delve deeper into the spatial differences among various histologic subtypes of early‐stage LUADs, we employed GeoMx digital spatial profiling (DSP) technology.^19^ The core objective of this study was to establish spatially resolved molecular profiles associated with tumour differentiation. This was achieved by systematically comparing transcriptomic profiles across histologic subtypes (well, moderately and poorly differentiated tumours) while accounting for cellular heterogeneity through compartment‐specific analysis of epithelial‐, macrophage‐ and stromal‐enriched regions. This approach simultaneously enabled the identification of regionally restricted biomarkers that reflect both differentiation gradients and prognostic outcomes in mixed‐type LUAD.

## 2 MATERIALS AND METHODS

### 2.1 Patient information

Tumour tissues from patients with early‐stage (stage I) LUADs were collected at Pathology Department of TianJin Chest Hospital from 2008 to 2020 (Table S1). All specimens underwent formalin fixation and paraffin embedding (FFPE) followed by sequential slicing, maintaining a slice thickness of 4–6 µm. Haematoxylin and eosin (H&E) staining was applied to the FFPE tissue sections to confirm distinct histologic subtypes by two experienced thoracic pathologists in blinded manner, including lepidic, acinar, papillary, micropapillary, solid adenocarcinoma and complex glandular adenocarcinoma patterns. These histologic subtypes were further divided into well, moderately and poorly differentiation grades. The pathological diagnosis of individual sections was performed according to the 2021 WHO classification of LUAD and the new grading system proposed by International Association for the Study of Lung Cancer pathology committee. This study has been approved by the Ethics Committee of Tianjin Chest Hospital and all participants provided signed informed consent. Additionally, 530 patients with LUAD were enrolled from The Cancer Genome Atlas (TCGA) (TCGA‐LUAD cohort).

### 2.2 Digital spatial profiling

GeoMx DSP (Nanostring) was performed according to previously published methods.^19^ Immunofluorescence assays were performed using antibodies against PanCK (Nanostring, catalogue #GMX‐RNA‐MORPH‐HST‐12), CD68 (sc‐20060 AF647, santa), CD163 (ab282114, abcam) and Syto13 (S7575, Invitrogen) for nuclear stain, and the multicolour images were used to guide the selection of regions of interest (ROIs) for each slide. For each ROI, areas of interest (AOIs) were extracted with image segmentation from the sequential masks as epithelial‐enriched (PanCK‐positive), macrophage‐enriched (CD68‐positive or CD163‐positive) and stromal‐enriched (PanCK‐negative, CD68‐negative, CD163‐negative) compartments. AOIs containing fewer than 100 nuclei were excluded. For each AOIs, index oligonucleotides of 18 677 RNA gene released from each AOI were collected into a 96‐well plate for quantification (GeoMx whole transcriptome atlas panel, Nanostring), libraries were prepared following NanoString's instructions and sequenced on a DNBSEQ‐T7 platform.

### 2.3 Spatial transcriptome data analysis

GeoMx NGS pipeline (v2.0.0.16) is used to convert sorted FASTQ files into DCC files. The DCC file is then uploaded to the GeoMx DSP system. Perform analysis using the data analysis module v.2.4.0.421 in the GeoMx DSP control centre. The QC of transcriptome data includes technical signals, technical background, probes and standardisation. When the alignment rate between the reading and the template sequence is less than 80%, use the technical signal QC to remove AOI. The technical background includes three indicators: template‐free control (NTC) count, negative probe count and AOI parameters. NTC counting is used to detect template contamination during library construction. AOIs with NTC greater than 1000 have been removed. In total, 128 AOIs were finally analysed (Table S2). In the Whole transcriptome analysis (WTA) experiment, negative probe counting is used to measure the overall technical signal level. The threshold for negative probe counting is four counts. Use 75th percentile normalisation to adjust the size of different AOIs to avoid differences between them. Hierarchical clustering and correlation matrix were performed using the ‘pHeatmap’ R package (v1.0.12). Principal component analysis (PCA) was performed using ‘prcomp of stats’ (v4.1.0). Related graphs were generated by the ‘ggplot2’ R package (v3.4.2).

### 2.4 Differential gene expression analysis

For differential expression genes analysis between two groups, edgeR (v3.42.4) was used, with a threshold |logFC| greater than 1 and *p*‐value <.05 to screen for differentially expressed genes (DEGs). Eight macrophage‐enriched and two stromal‐enriched AOIs were excluded due to high epithelial contamination. Epithelial contamination was assessed using the xCell algorithm (v1.1.0), with a score >.05 defined as the threshold (determined based on the distribution of epithelial scores in epithelial‐, macrophage‐ and stromal‐enriched AOIs, as all epithelial‐enriched AOIs >.05).

To rigorously identify genes exhibiting progressive upregulation (pattern 1), downregulation (pattern 2), highest in moderately (pattern 3) and lowest in moderately (pattern 4) across differentiation subtypes (well, moderately and poorly), we performed the Wilcoxon rank‐sum test to compare gene expression between each pair of groups (well vs. moderately, moderately vs. well, well vs. poorly). *p*‐Value <.1 in at least two pairwise comparisons were selected. Based on the logFC values, we categorised the genes into four patterns: (1) pattern 1 (progressive upregulation), logFC > 0 in all valid comparisons (e.g., well < moderately < poorly); (2) pattern 2 (progressive downregulation), logFC < 0 in all valid comparisons (e.g., well > moderately > poorly); (3) pattern 3 (moderately peaked), logFC > 0 in well versus moderately and logFC < 0 in moderately versus poorly (e.g., well < moderately > poorly); (4) pattern 4 (moderately troughed), logFC < 0 in well versus moderately and logFC > 0 in moderately vs. poorly (e.g., well > moderately < poorly).

### 2.5 Multiplex immunofluorescence staining

FFPE samples from 20 patients were subjected to multiplex immunofluorescence (mIF) staining, including 11 patients who underwent DSP sequencing and nine additionally recruited patients. The mIF staining was performed on unstained slides of FFPE specimens using the Opal 7‐Color Kits (Akoya Biosciences). Six markers were divided into two panels as follows: panel 1 included DAPI, pancytokeratin (epithelial marker), CD68 (macrophage marker), C3, CFB, CCL20 and C1R; panel 2 contained DAPI, pancytokeratin, CD68, MMP9, MUC5B, SPP1 and C1S. The stained slides were scanned using a Vectra 3.0 multispectral microscope system (Akoya Biosciences) at 10× magnification. Representative ROIs were selected using Phenochart 1.0.9 viewer (Akoya Biosciences) after comparing with H&E slides to capture malignant/premalignant cell clusters and heterogeneous elements. Corresponding normal ROIs were identified in morphologically normal tissues at the farthest tumour periphery on the same slide. Each ROI from panels 1 and 2 was aligned with sequential sections. Target areas were analysed using inForm 2.4.4 software (Akoya Biosciences). Subsequently, each ROI was partitioned into two compartments: the epithelial compartment and the tumour stroma compartment. Individual cells were identified by DAPI nuclear staining and co‐localisation markers.

### 2.6 Single‐cell RNA sequencing data processing and analysis

A single‐cell RNA sequencing (scRNA‐seq) dataset from 10 LUAD patients was obtained via the Code Ocean capsule (https://doi.org/10.24433/CO.0121060.v1). Patient histologies were classified into three categories: well differentiated (lepidic subtypes); moderately differentiated (acinar and papillary subtypes); and poorly differentiated (solid and mucinous subtypes). Quality control filtering excluded cells with fewer than 300 expressed genes, <3% ribosomal genes, >.1% haemoglobin genes, or >20% mitochondrial read counts. Genes detected in fewer than three cells were also removed. Data processing was performed using Seurat v5. Dimensionality reduction was conducted via PCA on the first 30 principal components (PCs), followed by uniform manifold approximation and projection (UMAP) for visualisation. Clustering was performed using FindClusters with a resolution of 1 on the first 30 PCs. Cell‐type annotation was carried out using established markers from the literature. Epithelial and malignant cells were isolated to compare the expression levels of *C1S*, *C1R* and *C3* across differentiation groups. Macrophage populations were extracted to analyse the expression of *MMP9*, *MALL* and *SUSD2* across differentiation groups.

### 2.7 Functional annotation and signature score analysis

For functional annotation, DEGs were processed using the clusterProfiler R package (v4.8.3). We used an adjusted *p*‐value of .05 as the statistical cutoff. Benjamini‒Hochberg procedure was used for adjusting *p*‐values to control for multiple testing. We computed signature scores via GSVA (v2.2.0) with ‘ssgsea’ method, using Molecular Signatures Database (MSigDB) hallmark gene sets.

### 2.8 Tumour microenvironment component estimation

CIBERSORT (v0.1.0)^20^ was used to estimate the proportion of immune subpopulations in tumour microenvironment (TME). Leveraging the LM22 reference gene signature provided by the software, we inferred cell‐type compositions from gene expression data using a digital deconvolution algorithm. The analysis was performed with parameters set to perm = 1000 and QN = TRUE. Samples were retained for statistical analysis only if they met the criteria of having a Monte Carlo permutation test *p* <.05 and a correlation coefficient >.7, ensuring robust and reliable deconvolution results.

### 2.9 Construction of a multi‐gene prognostic signature

To construct the 17‐gene prognostic signature, we integrated genes from three key pathways (complement activation, extracellular matrix [ECM] remodelling, humoural immune response) identified via spatial transcriptomic analysis. Genes were selected based on differential expression across histological grades and survival associations. Cox proportional hazards regression was applied to derive risk scores. The signature was further validated using Kaplan‒Meier (K‒M) analysis and receiver operating characteristic (ROC) curves. High‐ and low‐risk groups were defined by median risk scores. Independent validation was performed in the TCGA‐LUAD cohort (*n* = 530) using multivariate Cox regression and survival analysis. Statistical significance was set at *p* < .05.

### 2.10 Statistics analysis

Dimension reduction analysis was performed using umap (v0.2.8.0). Wilcoxon test was used to compare the differences between two groups. Correlation analysis was conducted using Pearson correlation analysis. The K‒M curve was compared using the log‐rank test. The median follow‐up time was calculated using the reverse K‒M method. Statistical significance was set at a *p*‐value of <.05.

## 3 RESULT

### 3.1 Spatial transcriptome profiling of tumour heterogeneity in mixed‐type early‐stage LUAD

**FIGURE 1:**
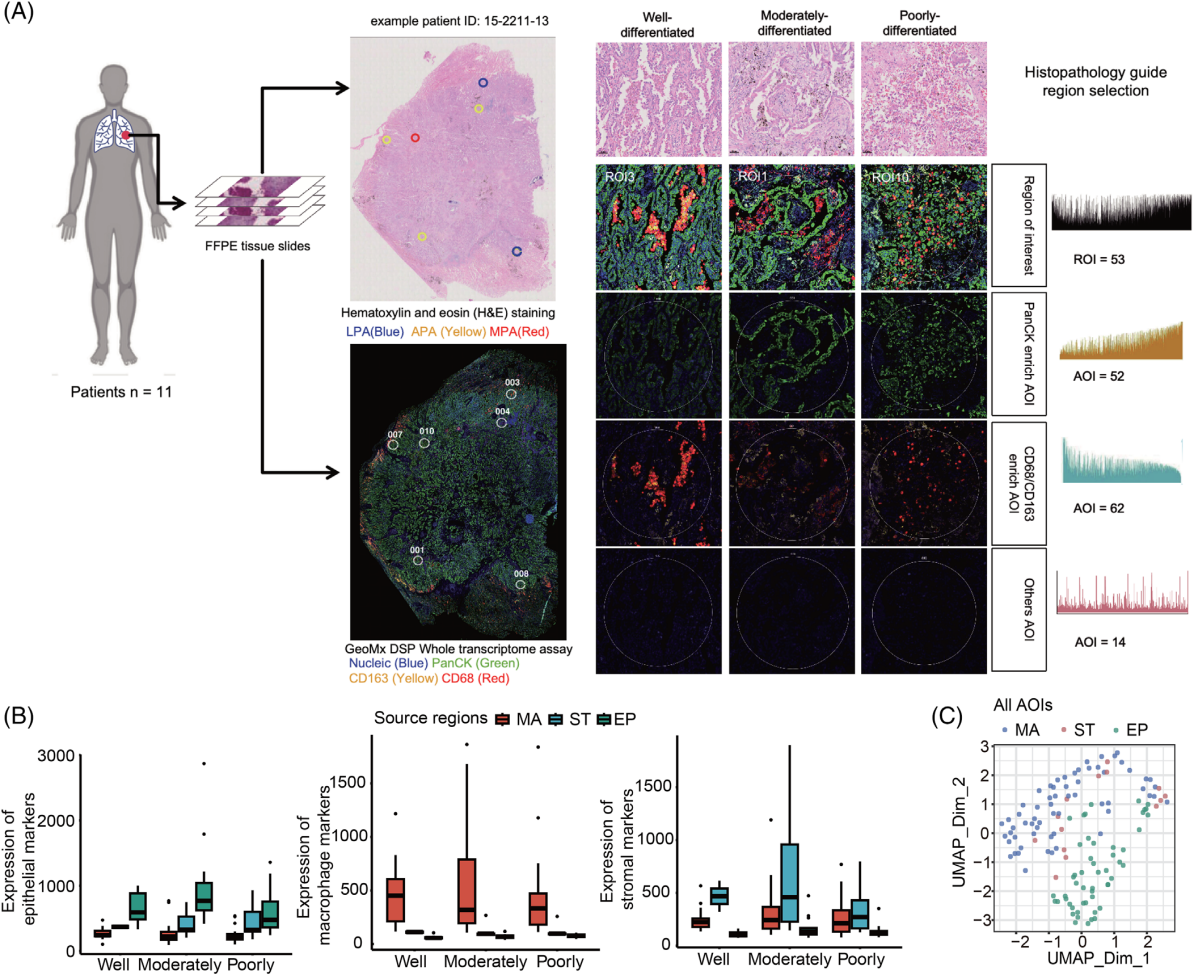
Digital spatial profiling (DSP) of early‐stage lung adenocarcinoma (LUAD) with mixed‐type histologic subtypes. (A) Overview of the study design for haematoxylin and eosin (H&E) and GeoMx DSP. (B) Marker genes distribution of three compartments at different subtypes of LUAD. (C) Uniform manifold approximation and projection (UMAP)‐based areas of interest (AOIs) from different compartments. EP, epithelial‐enriched compartments; MA, macrophage‐enriched compartments; ST, stromal‐enriched compartments.

To investigate the heterogeneity of the tumour immune microenvironment in patients with mixed‐type LUAD, this study analysed tumour tissues from 11 patients with primary early‐stage LUAD using spatial transcriptome profiling. FFPE tumour sections were prepared from each patient, and H&E staining was performed to identify histologic subtypes under the guidance of two experienced pathologists in a blinded manner, with each sample showing median three histologic subtypes (range 1–6) (Figure 1A and Table S1). Based on the differentiation grade of the histologic subtypes, we classified the histologic subtypes into three groups in accordance with the 2021 WHO classification of LUAD and the new grading system proposed by the International Association for the Study of Lung Cancer pathology committee: well differentiated (including lepidic pattern), moderately differentiated (including acinar or papillary patterns) and poorly differentiated (including micropapillary, solid and complex glandular patterns). To systematically characterise the molecular changes associated with the evolution of different histologic subtypes in mixed‐type LUAD, we performed spatial transcriptome profiling using GeoMx DSP platform. The GeoMx DSP process encompassed probe hybridisation with immunofluorescence antibodies, ROIs selection and segmentation, and transcriptome quantification through next generation sequencing. Immunofluorescence assays were performed using antibodies against PanCK, CD68, CD163 and Syto13, and the multicolour images were used to guide the selection of 53 ROIs from 11 samples. Each ROI was further subdivided into three types of AOIs through image segmentation, including epithelial‐enriched (PanCK+), macrophage‐enriched (CD68+ or CD163+) and others. The majority of the remaining areas were classified as stromal‐enriched (PanCK‒, CD68‒ and CD163‒) compartments (Table S2). After AOIs selection, the DNA oligonucleotides of 18 677 genes were UV‐cleaved and sequenced for mRNA quantitation, yielding 128 AOIs that met quality control criterion for subsequent analysis, including 29 well‐differentiated, 47 moderately differentiated and 52 poorly differentiated AOIs (Table S3).

Consistent with our segmentation strategy, significantly high expression of PanCK, CD68 or CD163, and stromal signatures was observed in epithelial‐, macrophage‐ and stromal‐enriched compartments, respectively (Figure 1B). Analysis of all spatially defined AOIs using UMAP‐based dimensionality reduction revealed a distinct separation between epithelial and TME compartments, as expected (Figure 1C). These results confirm the accurate segmentation and quantification of our ROIs using DSP technology, laying the foundation for more detailed exploration of the molecular signatures associated with distinct histologic subtypes of LUAD in subsequent analyses.

### 3.2 Molecular signatures and pathway dynamics in epithelial‐enriched compartments across histologic subtypes

**FIGURE 2:**
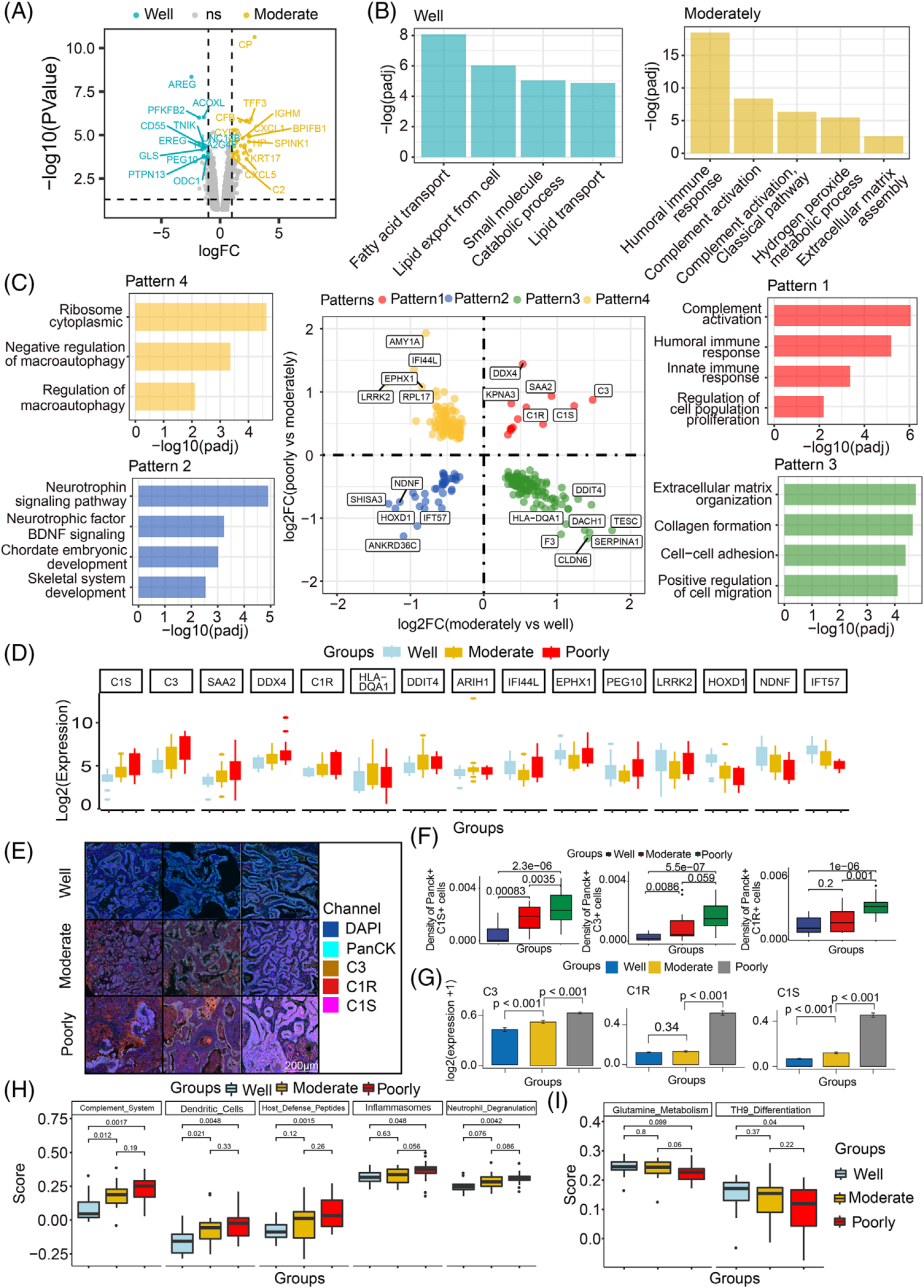
Changes in gene expression patterns in the epithelial‐enriched compartments among differentiation grades. (A) Volcano plot of significant differentially expressed genes between well and moderately differentiated groups. (B) Pathway enrichment analysis of differentially expressed genes between well and moderately differentiated groups. (C) The four‐quadrant diagram of gene expression patterns and related pathways among four different gene patterns during the histologic subtype progression. Genes in pattern 1 (marked in red) represent those continuously upregulated from well to poorly differentiation grade. Pattern 2 genes (blue) denote those continuously downregulated. Pattern 3 genes (green) exhibit upregulation from well to moderate differentiation followed by downregulation in poorly differentiated grade, whereas pattern 4 genes (yellow) show the opposite trend: downregulation from well to moderate differentiation followed by upregulation in poorly differentiated grade. (D) Gene expression with different patterns among differentiation groups. (E) Representative multiplex immunofluorescence (mIF) images showing the expression of C1S, C3 and C1R within PanCK‐positive cells across distinct tumour differentiation grades. Scale bars: 200 µm. (F) Boxplots comparing the density of C1S‐, C3‐ and C1R‐positive cells within the PanCK‐positive cell across distinct differentiation grades. (G) Boxplots showing the expression levels of C1S, C3 and C1R in epithelial cells across differentiation grades, analysed using the single‐cell dataset from Bischoff et al. (H) Signatures of gradual upregulation among differentiation grades. (I) Signatures of gradual downregulation among differentiation grades. Statistical tests for panels (F‒I) were performed using the Wilcoxon rank‐sum test.

To explore the core mechanisms that promote tumour differentiation, we characterised the various cellular compartments across LUAD initiation stages, examining their transcriptional patterns, enrichment functions and gene signatures. Initial investigation of the DEGs in epithelial‐enriched compartments transitioning from well to moderately differentiated states revealed dysregulation in several LUAD‐specific pathways (Figure 2A). Compared to well‐differentiated tumours, moderately differentiated tumours showed significant upregulation of pathways involved in humoural immune response, complement activation, response to oxygen levels and ECM organisation (Figure 2B). Consistent with previously research, our study also identified upregulation of genes (*CP*, *HP* and *CYBA*) associated with oxidative stress in moderately differentiated tumours.^10^ Top upregulated genes linked to humoural immune response, such as *BPIFB1* and *IGHM* may play an critical roles in tumour development.^21^, ^22^ In contrast, well‐differentiated groups exhibited overexpression of genes involved in metabolic process and fatty acid transport. Metabolic‐related genes such as *PFKFB2* and *ACOXL* were upregulated in this group, underscores the paramount significance of sustaining metabolic stability in tumour biology.^23^, ^24^ These results demonstrate differences in the activity of distinct biological functions during the early stages of LUAD progression, highlighting the complexity and heterogeneity of tumour development.

Joint interrogation of deregulated genes across the three differentiated epithelial‐enriched AOIs identified four distinct expression patterns (Figure 2C,D). Pattern 1 comprises genes that are under expressed in well‐differentiated tumours and gradually upregulated in moderately and poorly differentiated groups. Pathway enrichment analysis highlights that these deregulated genes are significantly enriched in complement activation, humoural immune response and regulation of cell population proliferation. Consistent with previous studies, complement activation related genes such as *C1R*, *C1S* and *C3* in this pattern may contribute to LUAD progression.^25^, ^26^ The genes involved in pattern 2 are expressed in well‐differentiated tumours and are progressively downregulated in moderately and poorly differentiated tumours, revealing the gradual downregulation of negative regulators of cellular de‐differentiation during the initiation progression of LUAD. Genes such as *NDNF* and *HOXD1* are associated with neurodevelopment and inhibit LUAD progression.^27^, ^28^ In pattern 3, genes associated with ECM organisation or cell adhesion are most highly expressed in moderately differentiated tumours. This might indicate that moderately differentiated tumour cells are more actively involved in proliferation and differentiation processes, and have more complex interactions with the TME. In contrast, well and poorly differentiated cells, which have stable or undifferentiated states adapted to specific microenvironment conditions, showed lower expression of these genes. For example, *SERPINA1*, a gene that regulates inflammation, ECM remodelling and cell signalling, has been reported to be associated with tumour cell proliferation.^29^ In pattern 4, we observed that some genes related to macroautophagy or ribosome are expressed at the lowest level in moderately differentiated tumours, but at a higher level in both well and poorly differentiation. Collectively, our analysis indicates that complement activation‐related genes are upregulated during LUAD progression, whereas genes associated with metabolic processes and neurodevelopment are suppressed during this process.

To validate the robustness of our main findings, we expanded our cohort to 20 LUAD patients (including 11 patient samples used for DSP detection and nine additionally collected patient samples) for mIF analysis (Table S4). Results showed that the expression levels of C3, C1R and C1S in PanCK+ cells gradually increased with elevated de‐differentiation status (Figure 2E,F). Additionally, we further collected public single‐cell datasets of different histopathological subtypes.^30^ Based on cell clustering and annotation results, we validated that the expression trends of these genes in tumour epithelial cells across different stages of tumour progression were consistent with our results (Figure 2G).

Signature analysis revealed gradual upregulation of innate immunity related signatures (the complement system, dendritic cells, host defense peptides, inflammasomes and neutrophil degranulation) alongside nuclear factor‐kappa B (NF‐κB) signalling, TH2 differentiation, angiotensin system and B/T‐cell signatures (Figures 2H and S1). These findings suggest an increasing activation of inflammatory and innate immune responses during tumour de‐differentiation. A gradual downregulation expression pattern was observed in pathways related to glutamine metabolism, TH9 differentiation, Treg differentiation and neuroendocrine function (Figures 2I and S2). Additionally, ECM remodelling, oxidative stress, epithelial‒mesenchymal transition (EMT), interleukin‐1 (IL‐1) signalling and IL‐2 signalling exhibited initial upregulation followed by downregulation (Figure S3). This pattern might reflect the dynamic adaptation of cancer cells to TME changes.

Analysis of distinct gene expression across histologic subtypes (lepidic, acinar, etc.) revealed that *HOXD1*, *NDNF* (neurodevelopmental related), and *IFT57*, *ANKRD36C* (ciliary/cytoskeletal regulators) showed progressive downregulation from lepidic to solid subtypes (*p* < .05), aligning with histological de‐differentiation (Figure S4). Signature analysis revealed significant enrichment of complement system activation, dendritic cell recruitment and host defense peptides in aggressive subtypes (*p* < .05), alongside downregulation of TH9 differentiation (*p* < .05; Figure S5).

### 3.3 Metabolic reprogramming of macrophages from well to moderately and poorly differentiated regions

**FIGURE 3:**
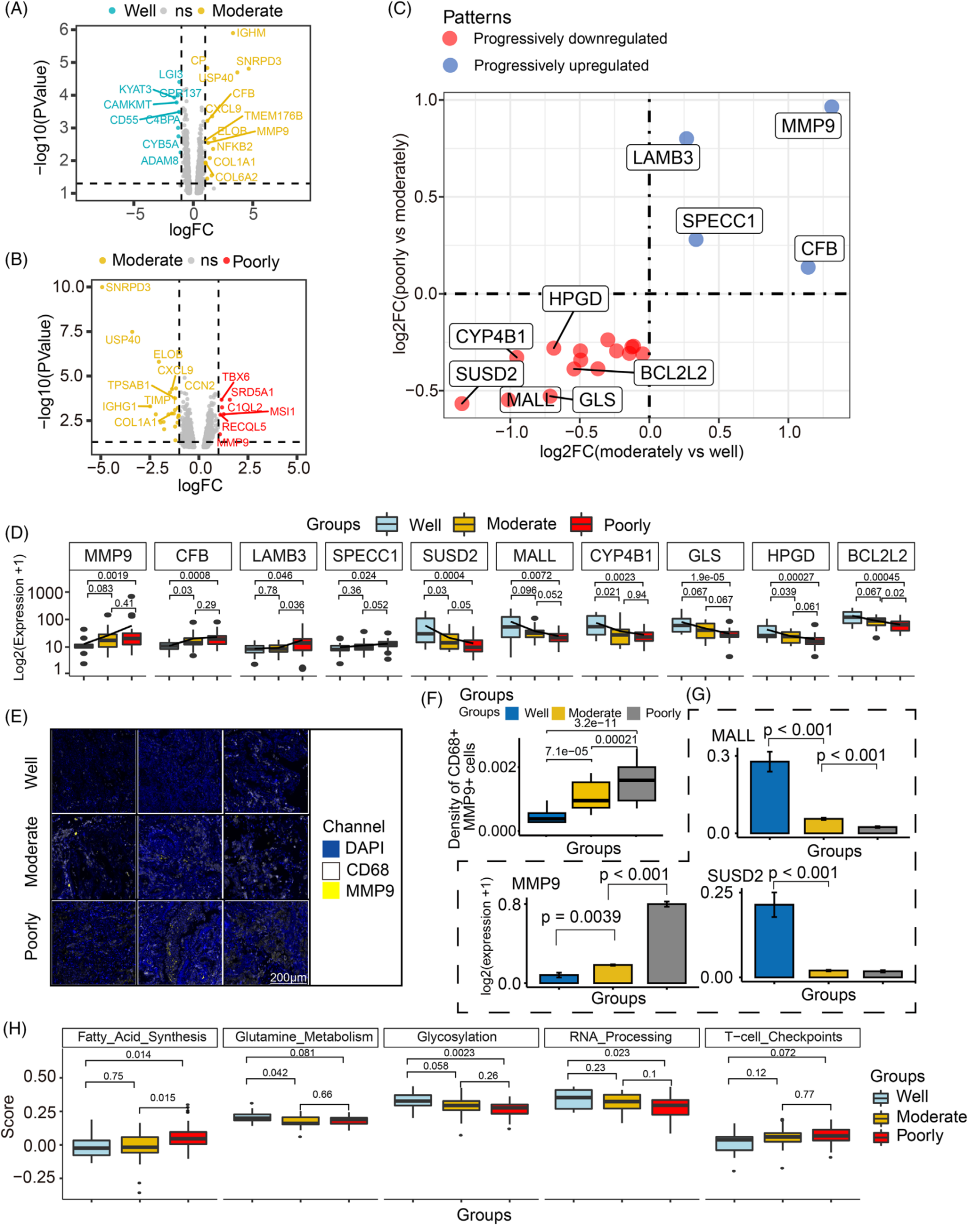
Changes in gene expression patterns in the macrophage‐enriched compartments among differentiation grades. (A) Volcano plot of differentially expressed genes (DEGs) between well and moderately differentiated groups. (B) Volcano plot of DEGs between moderately and poorly differentiated groups. (C) Genes with progressive upregulation or downregulation through the joint interrogation of deregulated genes across the three differentiated macrophage‐enriched areas of interest (AOIs). Genes that are progressively upregulated from well to poorly differentiated grades are marked in blue, whereas those that are progressively downregulated from well to poorly differentiated grades are marked in red. (D) Expression of genes with progressive upregulation or downregulation among differentiation groups. (E) Representative multiplex immunofluorescence (mIF) images showing the expression of MMP9 within macrophage‐enriched cells across distinct tumour differentiation grades. Scale bars: 200 µm. (F) Boxplots comparing the density of MMP9+ cells within macrophage cells among distinct differentiated grades. (G) Boxplots showing the expression levels of MMP9 in macrophage cells across differentiation grades, analysed using the single‐cell dataset from Bischoff et al. (H) Boxplot showing the comparison of signature scores among differentiation groups. Statistical tests for panels (F‒H) were performed using the Wilcoxon rank‐sum test.

Given the significant role of innate immunity, particularly complement activity, in tumour de‐differentiation observed in epithelial‐enriched AOIs, we next focused on the changes of macrophages, an important line of defense in innate immunity, across AOIs of varying differentiation status. We first conducted comparative analyses of macrophage‐enriched compartments in well‐, moderately and poorly differentiated samples. The results showed that metabolic process‐associated genes (*CYB5A* and *KYAT3*) were upregulated in the well‐differentiated group, which may influence macrophage polarisation and tumour suppression (Figure 3A).^31^ Moderately differentiated AOIs exhibited a transcriptome profile enriched in genes associated with collagen degradation and humoural immune response (Figure 3B). The upregulation of genes such as *MMP9* and collagen family suggests that increased collagen degradation might promote the migration of macrophage cells.^32^, ^33^ Poorly differentiated AOIs displayed a unique transcriptional profile characterised by high expression of genes related to humoural immune response, regulation of complement activation, proliferation and degradation of the ECM. Complement activation‐related genes, which are significantly overexpressed in the epithelial‐enriched compartment, was also prominently upregulated in macrophage‐enriched areas. In addition to that, ECM‐related gene such as *MMP9* and *MUC5B* were prominently upregulated in poorly group, indicating that the expression of these genes in macrophages plays a pivotal role in influencing tumour differentiation. To evaluate the association between spatially adjacent compartments, we analysed the correlation between complement genes in the epithelial‐enriched region and ECM‐related genes in the adjacent macrophage‐enriched region within the same ROI (*n* = 52). The results showed a significant positive correlation between complement genes (C3, C1S, C1R) in the epithelial‐enriched region and the ECM gene COL6A2 in the neighbouring macrophage‐enriched region (Spearman's *R* = .6, *p* < .01), with the correlation strength increasing as differentiation status decreases (Figure S6A). Additionally, the complement system signature was significantly positively correlated with the ECM remodelling signature (Spearman's *R* = .48, *p* = .032; Figure S6B). These findings support the hypothesis that tumour‒stroma crosstalk drives malignant progression via complement pathways.^34^

Next, we focused on genes that were progressively downregulated or upregulated across the well‐, moderately and poorly differentiated AOIs. As a result, we identified four genes that were progressively upregulated and 15 genes that were progressively downregulated (Figure 3C). Among these genes, *CFB* and *MMP9* emerged as the top two upregulated genes in the macrophage compartment, showing a progressive increase across differentiation stages (Figure 3D). In contrast, the metabolic‐related genes *GLS* and *CYP4B1* showed a progressive downregulation, exhibiting a gradual decrease in expression that mirrored the opposite trend (Figure 3D). Previous research suggests that *GLS* may activate macrophages by disrupting cellular metabolism.^35^ By comparing mIF results for MMP9 in macrophages across different groups, we observed an increasing trend in MMP9‐positive macrophages during the differentiation process, with a significant elevation in the poorly differentiated group (Figure 3E,F). Additionally, the findings regarding genes with significant changes in the macrophage‐enriched compartments (*MMP9*, *MALL* and *SUSD2*) were validated based on public scRNA‐seq datasets (Figure 3G).

Signature analysis further confirmed these transcriptional differences (Figure 3H). Gradient analysis revealed a decrease in glycosylation, glutamine metabolism and RNA processing signatures, along with an increase in fatty acid synthesis signature, in macrophage‐enriched areas from well to poorly differentiated AOIs. These biological functions have been demonstrated to be associated with the metabolic reprogramming of tumour‐associated macrophages (TAMs) within the TME.^31^ Additionally, the upregulation of T‐cell checkpoints in macrophage‐enriched areas was also observed, further suggesting tumour immune evasion and the formation of an immunosuppressive microenvironment at the poorly differentiated stage.

Analysis of gene expression patterns across different histological subtypes in macrophage‐enriched regions revealed progressive downregulation of metabolic genes (including *GLS*, *HPGD* and *MTR*) across histological subtypes (*p* < .05). Conversely, *MMP9* (matrix remodelling) and *CFB* (complement activation) were significantly upregulated (*p* < .05), highlighting enhanced ECM degradation and immune modulation in poorly differentiated subtypes (Figure S7). Signature analysis revealed a near‐significant decline in glycosylation metabolism (*p* = .07), indicating altered post‐translational regulation in macrophage populations within poorly differentiated niches (Figure S8).

### 3.4 Deciphering immune cell composition shifts within tumour microenvironment

**FIGURE 4:**
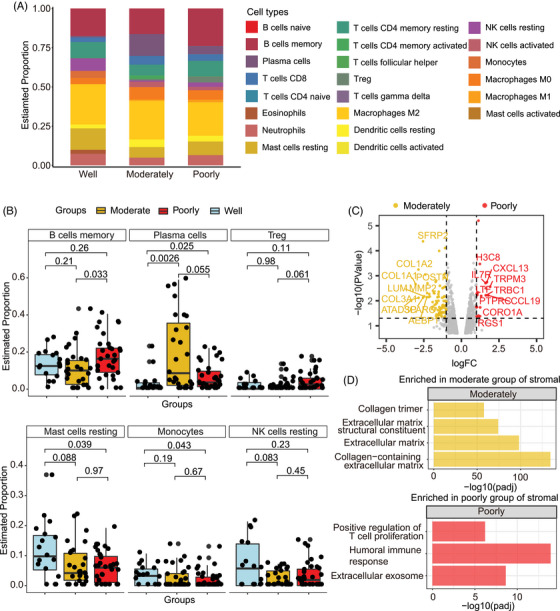
Immune cells and gene expression profiling in the tumour microenvironment (TME)‐enriched compartments among differentiation grades. (A) Immune cell types proportion across distinct differential grades estimated by cibersort. (B) Comparison of immune cells proportion in distinct groups. (C) Volcano plot of differentially expressed genes (DEGs) between moderately and poorly differentiated groups. (D) Pathway enrichment analysis of DEGs between moderately and poorly differentiated groups in TME‐enriched compartment. Statistical tests for panels (B) were performed using the Wilcoxon rank‐sum test.

Given that tumour initiation and progression are heavily shaped by their microenvironments, we further investigated the composition of immune cells to characterise TME changes during LUAD initiation. We initially quantified the immune cell types subsets in PanCK‐negative regions (Figure 4A). Compared to well‐differentiated AOIs, moderately differentiated AOIs showed upregulation of plasma cells and downregulation of resting natural killer (NK) cells and resting mast cells (Figure 4B). Furthermore, poorly differentiated AOIs, relative to moderately differentiated ones, exhibited upregulation of memory B cells and Tregs (Figure 4B). These findings suggest a correlation between decreased tumour differentiation and an enhanced immunosuppressive microenvironment. The observed increase in immunosuppressive cell populations such as plasma cells and Tregs, along with a decrease in potentially cytotoxic immune cells such as NK cells and mast cells, might contribute to the aggressiveness of less differentiated tumours by impairing anti‐tumour immune response.

Given the limited availability of well‐differentiated AOIs in the stromal‐enriched compartments, we focused our analysis on the differences between moderately and poorly differentiated tumours. In moderately differentiated stromal‐enriched regions, 80 genes were regulated (Figure 4C), which are primarily involved in maintaining the structural integrity and normal physiological functions of moderately differentiated stromal cells. For example, the collagen‐related genes (*COL1A1*, *COL1A2*, *COL3A1*) are crucial for ECM formation, which is essential for cell adhesion, migration and tissue organisation (Figure 4D).^32^ In contrast, poorly differentiated AOIs exhibited upregulation of 22 genes, with the highest fold change observed in immune regulatory marker genes (*CCL19*, *CXCL13* and *LTF*) (Figure 4D). These results highlight a bidirectional shift from stromal‐ECM dominance in moderate differentiation to immune‐inflammatory dysregulation in poorly differentiation.

### 3.5 Transcriptomic patterns associated with recurrence status as potential indicators for clinical prognosis

**FIGURE 5:**
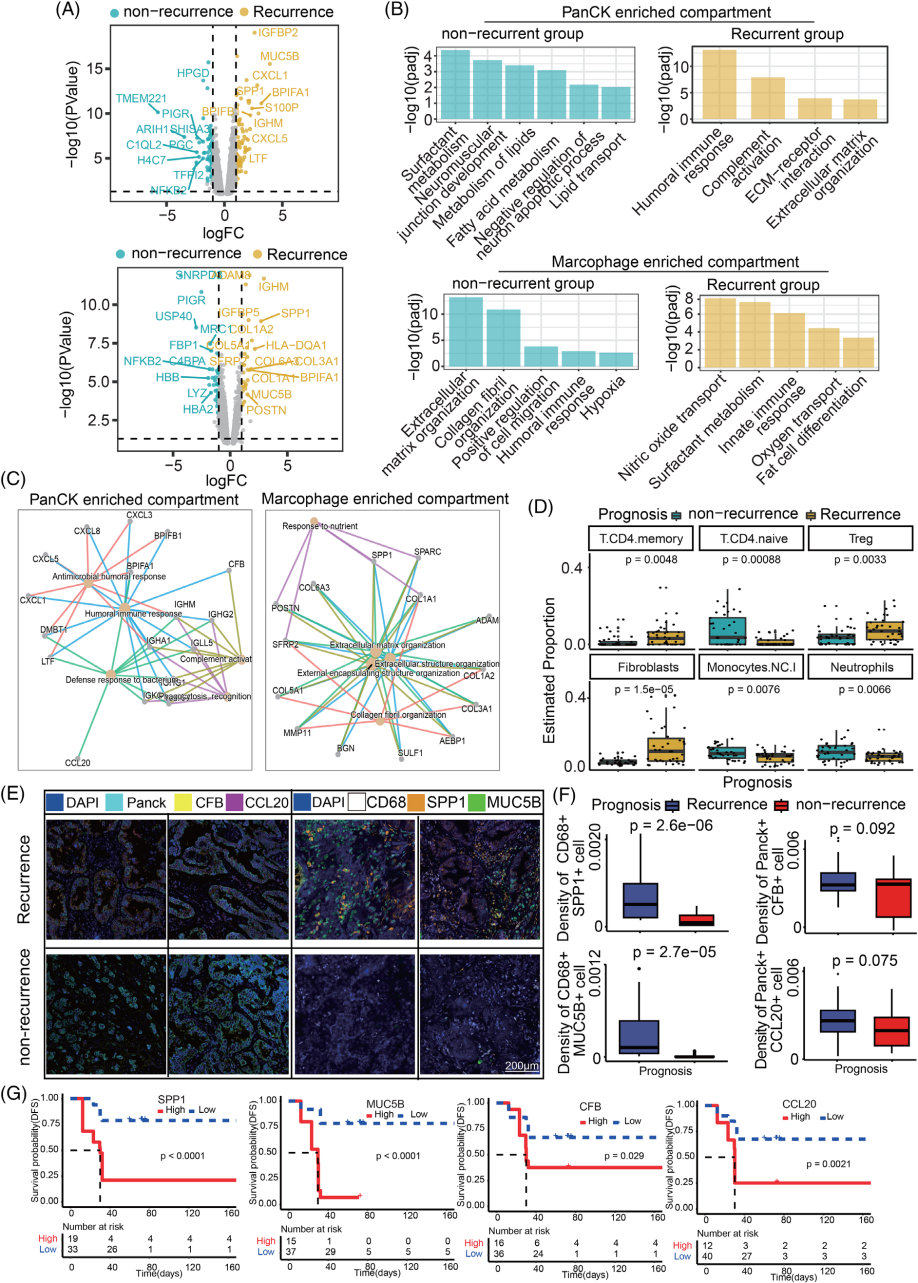
Gene expression patterns among recurrence and non‐recurrence groups. (A) Volcano plot of differentially expressed genes (DEGs) in epithelial‐enriched (above) and macrophage‐enriched (below) compartments between recurrence and non‐recurrence groups. DEGs in the non‐recurrence group are labelled in light blue; DEGs in the recurrence group are labelled in yellow. (B) Pathway enrichment analysis of DEGs. (C) DEGs involved in pathways enriched within PanCK‐positive and macrophage‐enriched compartments. (D) Comparison of immune cell proportions across different prognosis groups. (E) Representative multiplex immunofluorescence (mIF) images showing the expression of CFB and CCL20 within PanCK‐positive cells across distinct prognosis groups (left panel). Representative mIF images showing the expression of SPP1 and MUC5B within CD68‐positive cells across distinct prognosis groups (right panel). Scale bars: 200 µm. (F) Boxplots comparing the density of SPP1‐ and MUC5B‐positive cells within the CD68‐postivite cells and CFB‐ and CCL20‐positive cells within the PanCK‐positive cells across distinct prognosis groups. (G) The gene expression levels on disease progression free stratification. Statistical tests for panels (D and F) were performed using the Wilcoxon rank‐sum test. Statistical tests for panel (G) were performed using log‐rank test.

To gain a comprehensive understanding of the molecular underpinnings of recurrence in early‐stage LUAD, we conducted analysis on tumour samples between recurrent and non‐recurrent patient groups. In the epithelial‐enriched compartment, we identified a set of DEGs that were significantly associated with recurrence, such as *BPIFA1*, *BPIFB1* and *SPP1* (Figure 5A). These genes were enriched in pathways related to humoural immune response, complement activation, NF‐κB signalling pathway, ECM‒receptor interaction and focal adhesion, suggesting their potential roles in promoting tumour recurrence (Figure 5B). Additionally, signature analysis revealed associations between complement system, NF‐κB signalling, *TNF* and *p53* signatures with disease progression (Figure S9A). Functional annotation highlighted the role of these genes in oncogenic processes such as humoural immune response and complement activation (Figure 5C).

In the macrophage‐enriched compartment, we observed a distinct set of DEGs that were associated with disease outcome, such as *COL1A2*, *COL3A1*, *COL1A1*, *ADAM8* and *SPP1* gene (Figure 5A). Notably, *SPP1* was also among the top 10 significantly upregulated genes in epithelial‐enriched compartment. These genes were enriched in pathways related to ECM (Figure 5B). Signature analysis identified macrophage subsets with high expression of cell adhesion and innate immunity in recurrence group (Figure S9B). Functional annotation of macrophage‐specific DEGs implicated ECM in orchestrating tumour‒immune interactions (Figure 5C).

We analysed the correlations between PD‐L1 and three pathways (ECM remodelling, complement activation and humoural immune response) in epithelial‐ and macrophage‐enriched compartments. Weak negative correlations were observed between ECM remodelling and PD‐L1 in both tumour (*R* = ‒.28, *p* = .044) and macrophage regions (*R* = ‒.26, *p* = .039), as well as between complement activation and PD‐L1 in tumour regions (*R* = ‒.24, *p* = .08) (Figure S10). This finding aligns with emerging evidence indicating that LUAD might reshape the immune microenvironment through complement activation and ECM remodelling, potentially driving immune evasion via PD‐L1‐independent mechanisms.^36^, ^37^, ^38^ Analysis of TCGA data revealed weak negative correlations between the humoural immune response, complement activation and ECM remodelling pathways and tumour mutation burden (*p* < .001; Figure S11). This finding uncovers the intricate relationship between the TME and genomic instability.

Spatial profiling of immune cells in both epithelial and macrophage compartments revealed distinct immune landscapes in recurrent and non‐recurrent tumours. Specifically, recurrent tumours exhibited a higher abundance of immunosuppressive cell types, such as Treg‐ and myeloid‐derived suppressor cells, whereas non‐recurrent AOIs displayed a more favourable immune microenvironment with a higher proportion of effector T cells and NK cells (Figure 5D). To further validate these findings, we stratified the mIF results by prognosis (Figure 5E). Our analysis revealed an increasing trend in CFB‐ and CCL20‐positive cells within Panck+ populations in the recurrence group. In contrast, SPP1‐ and MUC5B‐positive macrophages were significantly elevated in the recurrence group (Figure 5F).

Survival analysis based on the transcriptional profiles of epithelial‐ and macrophage‐enriched compartments revealed significant differences in prognosis between recurrent and non‐recurrent tumours. Specifically, patients with tumours exhibiting high expression of ECM‐related (*SPP1* and *MUC5B*) and complete active related genes (*CFB* and *CCL20*) in either the epithelial or macrophage compartment had significantly shorter disease‐free survival (DFS) (Figure 5G). To validate our findings, we analysed survival data from TCGA‐LUAD cohort. Consistent with our results, tumours exhibiting similar transcriptional signatures in epithelial and macrophage compartments showed significantly different survival outcomes, further corroborating the prognostic value of our spatial transcriptomic analysis (Figure S12). Our transcriptomic analysis has confirmed prior reports that elevated expression of *SPP1* in both tumour and macrophage compartments is associated with poor prognosis in LUAD.^39^, ^40^ Furthermore, we extend this observation by revealing a significant correlation between *SPP1* overexpression and recurrence in mixed‐subtype LUAD, thereby highlighting its potential as a biomarker for disease recurrence and poor outcome in this heterogeneous patient population.

### 3.6 A multi‐gene model based on differentiation‐associated signatures with prognostic value

To address the limitations of single‐gene prognostic models, we systematically evaluated the prognostic value of differentiation‐associated signatures identified in epithelial‐ and macrophage‐enriched compartments (Figure S13). In the epithelial‐enriched compartment, the complement activation signature exhibited a significant negative correlation with DFS (*p* = .012), while lipid metabolism (*p* < .001) and glycolysis/glucose transport metabolism signatures (*p* < .001) were positively associations with DFS. In the macrophage‐enriched compartment, the complement activation (*p* = .02) and ECM remodelling signatures (*p* < .001) were inversely correlated with DFS, whereas metabolic signature showed a protective association with improved survival outcomes (*p* = .02). These findings underscore the compartment‐specific roles of molecular pathways in shaping clinical trajectories.

**FIGURE 6:**
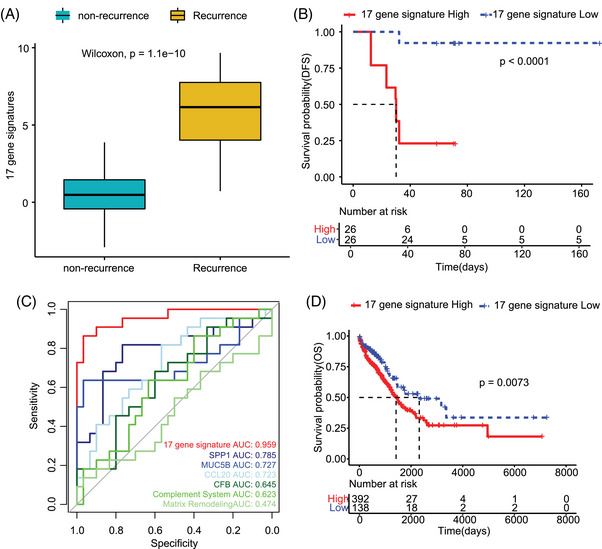
Seventeen gene signature score associated with prognostic of mixed‐type histological lung adenocarcinoma (LUAD). (A) Distribution of 17 gene signature scores in recurrence and non‐recurrence groups. (B) Disease‐free survival (DFS) difference between high‐ and low‐17 gene signature score. (C) Receiver operating characteristic (ROC) curves of efficacy prediction of constructed 17 gene signature, single genes and differentiation associated signatures. (D) Overall survival difference between high‐ and low‐17 gene signature score in The Cancer Genome Atlas (TCGA)‐LUAD cohort. Statistical tests for panel (A) were performed using the Wilcoxon rank‐sum test. Statistical tests for panels (B and D) were performed using log‐rank test.

To capture synergistic interactions between pathways and better reflect the biological complexity of LUAD progression, we constructed an integrated signature to identify high‐risk survival subgroups (Figure 6A). The 17‐gene signature developed based on Cox proportional hazards regression, incorporates genes from three key pathways: complement activation (*CFB*, *IGHA1*, *IGHG*, *IGHG2*, *IGHG4*, *IGHM*), ECM remodelling (*COL6A2*, *MMP1*, *SPP1*, *TNC*, *P3H3*) and humoural immune response (*BPIFA1*, *CXCL1*, *CXCL3*, *CXCL5*, *CXCL8*, *DMBT1*) (Table S5). K‒M analysis stratified patients into high‐ and low‐risk groups based on median signature scores, revealing significantly shorter DFS in high‐risk patients (*p* < .0001; Figure 6B). The prognostic superiority of this signature was confirmed by ROC analysis, which yielded an area under the curve (AUC) of .96 and significantly outperform individual genes and existing signatures (AUC range: .47–.79; Figure 6C). Independent validation in the TCGA‐LUAD cohort (*n* = 530) further demonstrated its generalisability, with high‐risk patients exhibiting significantly reduced overall survival (Hazard ratio = 1.62, *p* = .0073; Figure 6D). This multi‐gene model highlights the synergistic prognostic value of spatially resolved pathway interactions in LUAD.^34^, ^41^

In summary, our spatial transcriptomic analysis provides novel insights into the molecular mechanisms underlying tumour recurrence in early‐stage LUAD. The identified prognostic markers and signatures offer potential targets for therapeutic intervention and disease monitoring.

## 4 DISCUSSION

In the current study, we explore the intricate differences between pathological tissue subtypes of early‐stage LUAD, aiming to elucidate molecular mechanisms underlying tumour malignancy progression and identify prognostic biomarkers. By utilising GeoMx DSP and mIF to analyse molecular expression separately from epithelial‐, macrophage‐ and stromal‐enriched regions within the same sample, we gained novel insights into the heterogeneity and complexity of these tumours.

Our analysis revealed shared characteristics between epithelial‐ and macrophage‐enriched AOIs during the progression from well‐differentiated to moderately and poorly differentiated adenocarcinoma. The enrichment of neurodevelopmental of tumour core and metabolic pathways of macrophage in well‐differentiated tumours suggests that these cells might rely more on developmental cues and metabolic reprogramming for growth and maintenance. However, the most intriguing findings emerged in poorly differentiated tumours, where pathways related to humoural immune response, regulation of complement activation and ECM‒receptor interaction pathways were enriched. These pathways are typically associated with immune surveillance and TME remodelling,^42^, ^43^, ^44^, ^45^, ^46^ but in the context of cancer, they might indicate a more aggressive phenotype.^47^, ^48^, ^49^, ^50^ The enrichment of humoural immune response genes suggests an increased presence of antibodies or other humoural factors, which could be involved in tumour‐associated immune responses. The regulation of complement activation and ECM‒receptor interaction pathways further supports the idea that poorly differentiated tumours are actively remodelling their microenvironment to facilitate invasion and metastasis. The association between these immune and ECM‐related pathways and worse prognosis is particularly noteworthy.^51^, ^52^ This suggests that targeting these pathways could potentially improve therapeutic outcomes in patients with poorly differentiated LUAD. For example, therapies that modulate the immune response or disrupt ECM‒receptor interactions could be explored as potential treatment strategies.

We identified a crucial role of genes related to the complement system in epithelial cells and macrophages as potential drivers of malignant progression in LUAD. This finding adds to the complexity of the complement system, which is traditionally viewed as a key component of innate immunity and anti‐tumour defense but has also been shown to facilitate tumour growth, angiogenesis, modulate the TME and enhance tumour cell metastasis.^53^ Our results demonstrate the upregulation of *C1R*, *C1S*, *C3*, *IGHM* and *CFB* in poorly differentiated tumours, suggesting activation through the classical pathway. To validate the expression patterns of these genes, we performed mIF experiments and cross‐referenced public scRNA‐seq datasets. Recent research has highlighted the role of tumour‐derived *C3* in promoting tumour growth via intracellular activation and interaction with T cells.^54^ Additionally, *C3* modulates the polarisation of TAMs, suppressing anti‐tumour immune responses.^55^ These findings are supported by Talaat et al.,^56^ who reported that *C3* participates in EMT through the activated C3a fragment, inducing E‐cadherin expression and thereby enhancing tumour invasiveness. The negative regulatory effect of complement activation in the TME suggests a novel therapeutic target for immunotherapy.^57^, ^58^ Tumour cell‐derived complement proteins, such as *C3*, which inherently inhibit anti‐tumour immunity, could potentially serve as effective targets for cancer immunotherapy, enhancing the anti‐tumour immune capabilities of the complement system. The identification of these mechanisms offers a rationale for developing novel therapeutic strategies targeting the complement system to augment anti‐tumour immunity and potentially improve patient outcomes. However, further research is needed to fully understand the intricacies of the complement system in cancer and to develop effective therapeutic interventions. The role of complement components in cancer is complex and multifaceted, requiring a comprehensive understanding of their interactions with other components of the immune system and the TME. With this knowledge, we may be able to harness the anti‐tumour potential of the complement system while minimising its protumourigenic effects.

The ECM is a complex network of proteins and polysaccharides that provides structural support to tissues and cells.^58^, ^59^ In the context of cancer, alterations in the ECM are known to play crucial roles in tumour invasion, metastasis and drug resistance.^60^ The enrichment of ECM‐related genes in poorly differentiated tumours suggests that these cells might be undergoing a more aggressive transformation, actively remodelling their surrounding microenvironment to facilitate tumour growth and dissemination. One possible explanation for this finding is that poorly differentiated cancer cells might be more dependent on ECM components for anchorage and survival. We observed a consistent upregulation of *MMP9*, a finding that aligns with previous studies reporting its increase in solid LUAD.^61^ Notably, *MMP9* expression was found to be significantly higher in macrophage‐enriched compartments compared to the epithelial‐enriched compartments, displaying a gradient pattern that varied with tumour differentiation. This observation underscores the uniqueness of spatial transcriptomics compared to bulk sequencing approaches. Previous studies have identified MMP9+ macrophages as terminally differentiated TAMs, which are associated with tumour progression.^62^ Our data further support the involvement of *MMP9* and its macrophage‐derived forms in the complex regulatory networks governing lung cancer development and progression. In addition to that, ECM‐related genes such as *COL1A2*, *COL3A1* and *COL1A1*, might help these cells establish a favourable niche by promoting cell‒matrix interactions and signalling pathways that support proliferation and invasion. Importantly, the identification of ECM‐related genes as differentially expressed in poorly differentiated tumours provides potential new targets for therapeutic intervention. Moreover, understanding the role of ECM in tumour heterogeneity may help develop more personalised treatment strategies that target‐specific tumour subtypes.

Our integrated 17 gene signature underscores the critical role of pathway crosstalk in LUAD progression. By combining complement activation, ECM remodelling and humoural immunity, our model captures the multifaceted nature of tumour‒immune‒stromal interactions. The signature's AUC of .96 surpasses prior single‐gene markers (e.g., *SPP1*, *CFB*), suggesting enhanced clinical utility. Independent validation in TCGA‐LUAD reinforces its generalizability, positioning it as a potential tool for risk stratification in early‐stage disease. Mechanistically, the synergistic upration of complement and ECM genes might create a niche by facilitating immune escape, enhance tumour cell adhesion, migration and proliferation.

However, it is important to acknowledge the limitations of our study. The relatively small sample size may limit the generalisation of our findings. Larger‐scale multicentre studies are needed to validate and extend our observations. Our current work did not extensively apply advanced spatial statistics, primarily due to the pre‐selected ROI design of GeoMx DSP, which focuses on targeted regions, and its multicellular resolution, typically requiring more than 100 cells per AOI, rather than single‐cell resolution. In the future, we plan to integrate single‐cell resolution spatial analysis platforms (e.g., CosMx SMI or 10× Xenium). This will enable us to obtain more accurate cell types, perform co‐localisation analysis, neighbourhood patterning and develop spatial network models. Additionally, while the spatial transcriptomic analysis provided valuable insights, LUAD is a complex disease influenced by multiple factors, including genetic mutations and protein expression. Future studies should aim to integrate these various factors to provide a more comprehensive understanding of this malignancy.

## 5 CONCLUSION

In conclusion, our spatial transcriptomic analysis revealed significant differences between histologic subtypes of early‐stage LUAD. These findings enhance our understanding of the molecular mechanisms underlying this heterogeneous disease and might pave the way for more precise diagnostic and therapeutic approaches. With further research, we hope to translate these insights into clinical practice and improve patient outcomes.

## AUTHOR CONTRIBUTIONS

*Conception and design*: Hua Geng, Meilin Xu and Congcong Song. *Collection and assembly of data*: Hua Geng, Meilin Xu, Shixiong Li and Yujie Zhao. *Data analysis and interpretation*: Hua Geng, Wenhao Zhou, Yujie Zhao and Congcong Song. *Manuscript writing*: Hua Geng, Jiaqian Wang and Haitao Luo. All authors contributed to subsequent drafts and made the decision to submit the report for publication.

## CONFLICT OF INTEREST STATEMENT

The authors declare they have no conflicts of interest.

## ETHICS STATEMENT

Ethics Committee of Tianjin Chest Hospital gave its approval to the project (ethics approval no. 2025LW‐10).

## Supporting information



Supporting Information

Supporting Information

Supporting Information

Supporting Information

Supporting Information

Supporting Information

Supporting Information

Supporting Information

Supporting Information

Supporting Information

Supporting Information

Supporting Information

Supporting Information

Supporting Information

Supporting Information

Supporting Information

Supporting Information

Supporting Information

## Data Availability

The original contributions presented in the study are included in the Supporting Information, further inquiries can be directed to the corresponding author.
