# Supplementary figures and images for: Spatial transcriptomic analysis across histological subtypes reveals molecular heterogeneity and prognostic markers in early‐stage lung adenocarcinoma
Source: Clin Transl Med. 2025 Aug 22;15(8):e70439. doi: 10.1002/ctm2.70439 (PMC12373976; doi:10.1002/ctm2.70439)

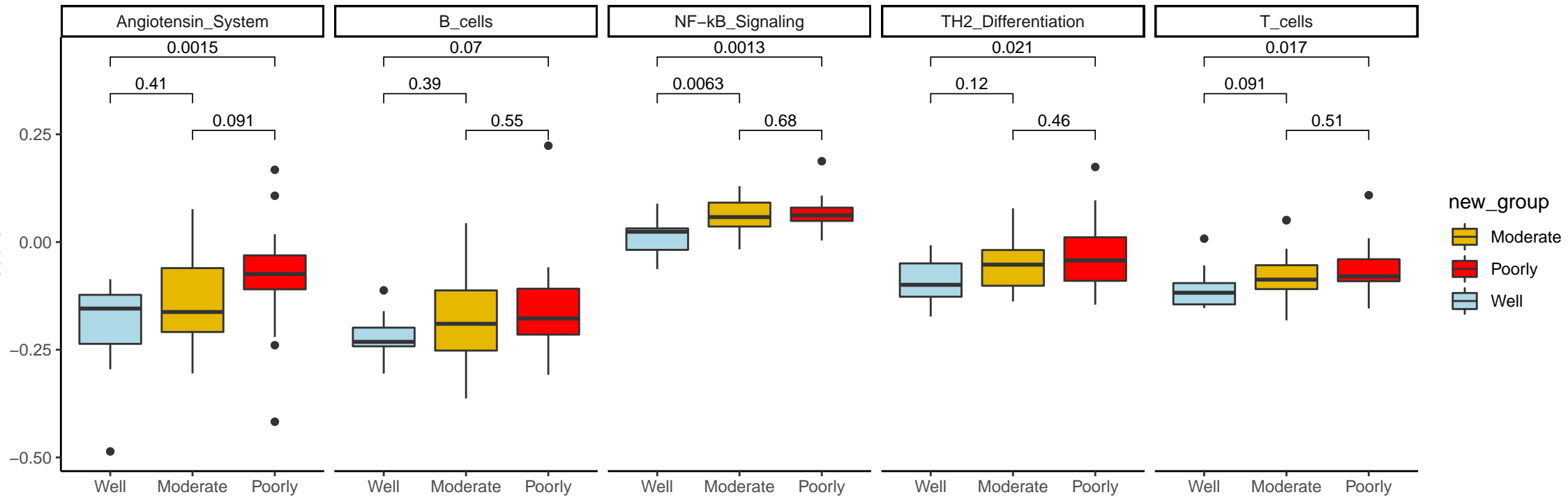

Supplement: Supplementary file 1 — Supporting Information [file CTM2-15-e70439-s018.pdf]

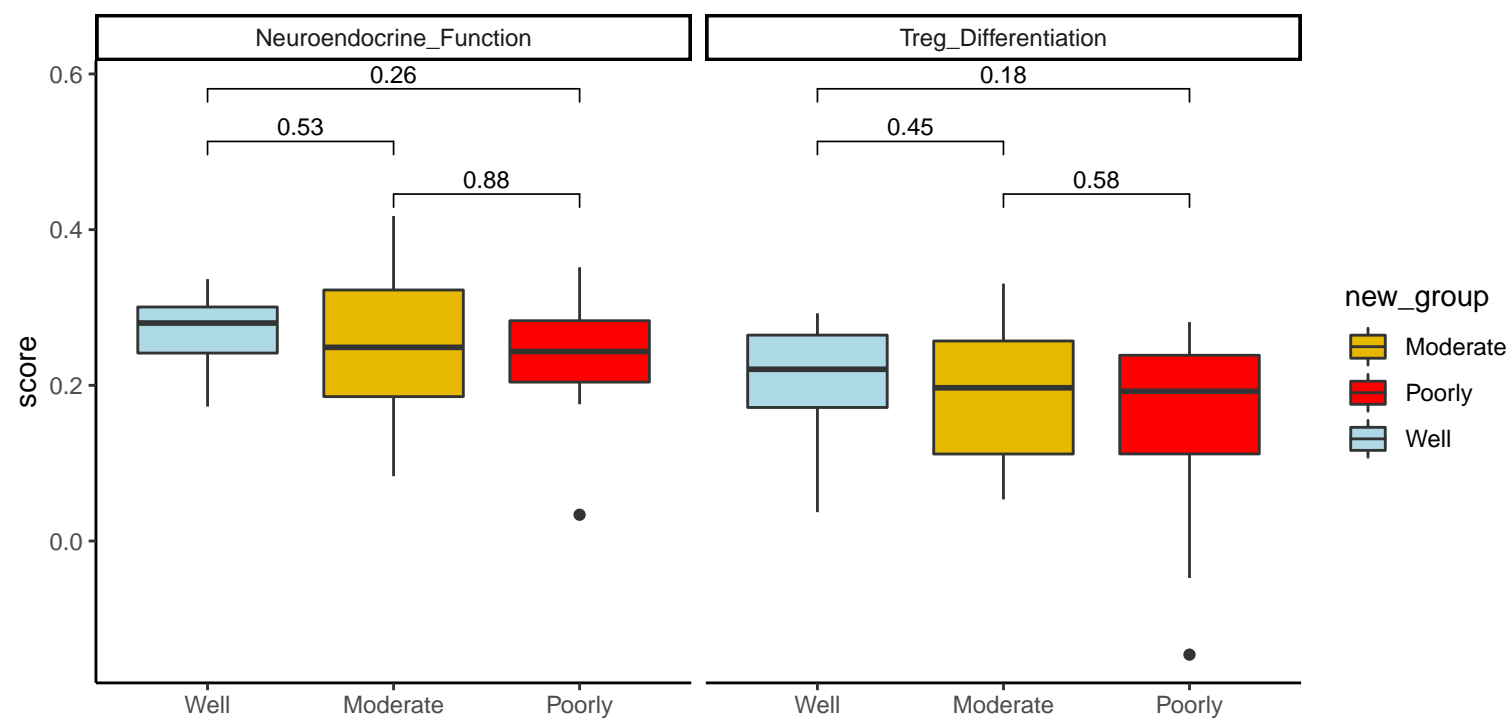

Supplement: Supplementary file 2 — Supporting Information [file CTM2-15-e70439-s016.pdf]

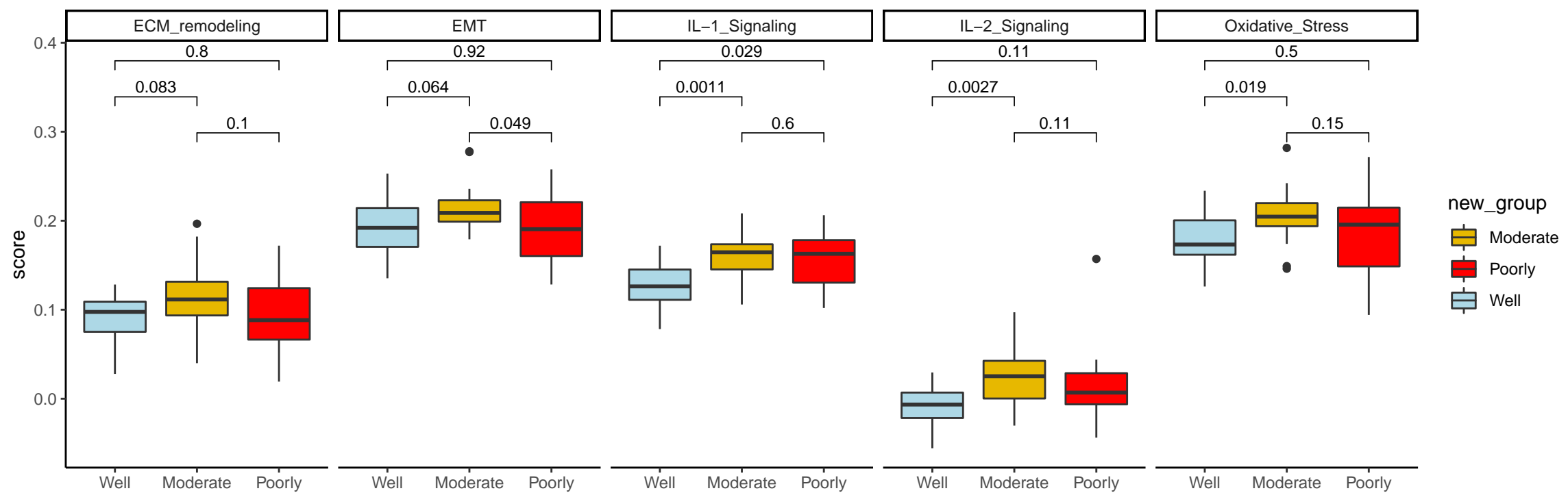

Supplement: Supplementary file 3 — Supporting Information [file CTM2-15-e70439-s008.pdf]

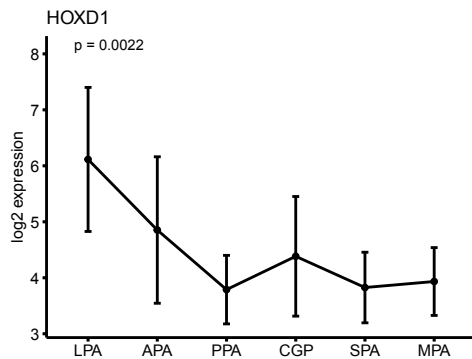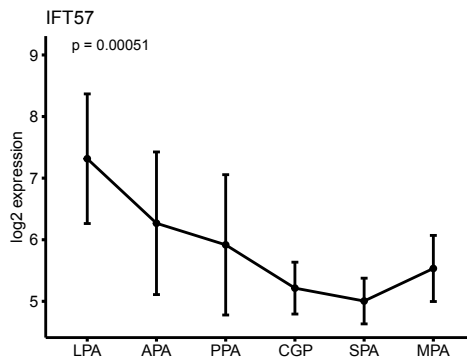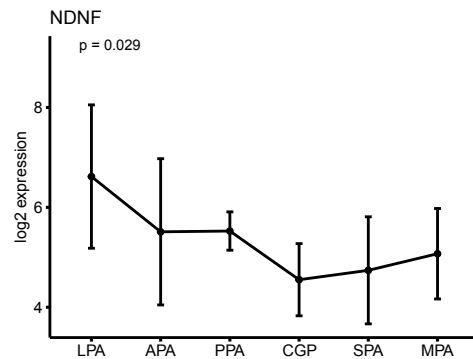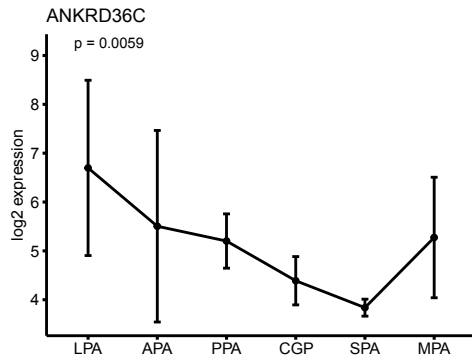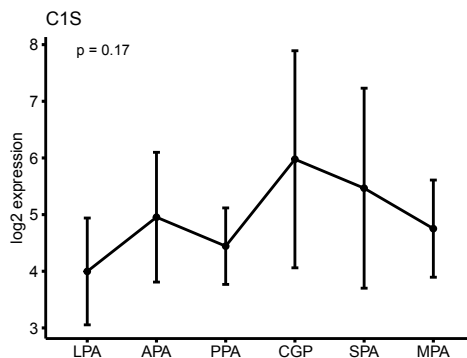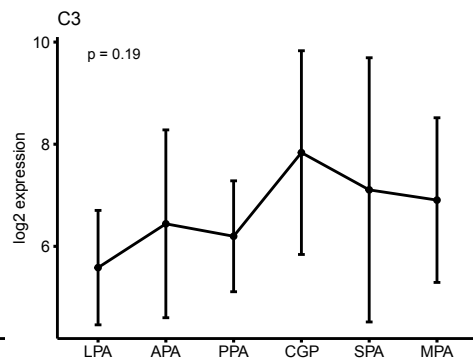

Supplement: Supplementary file 4 — Supporting Information [file CTM2-15-e70439-s003.pdf]

score

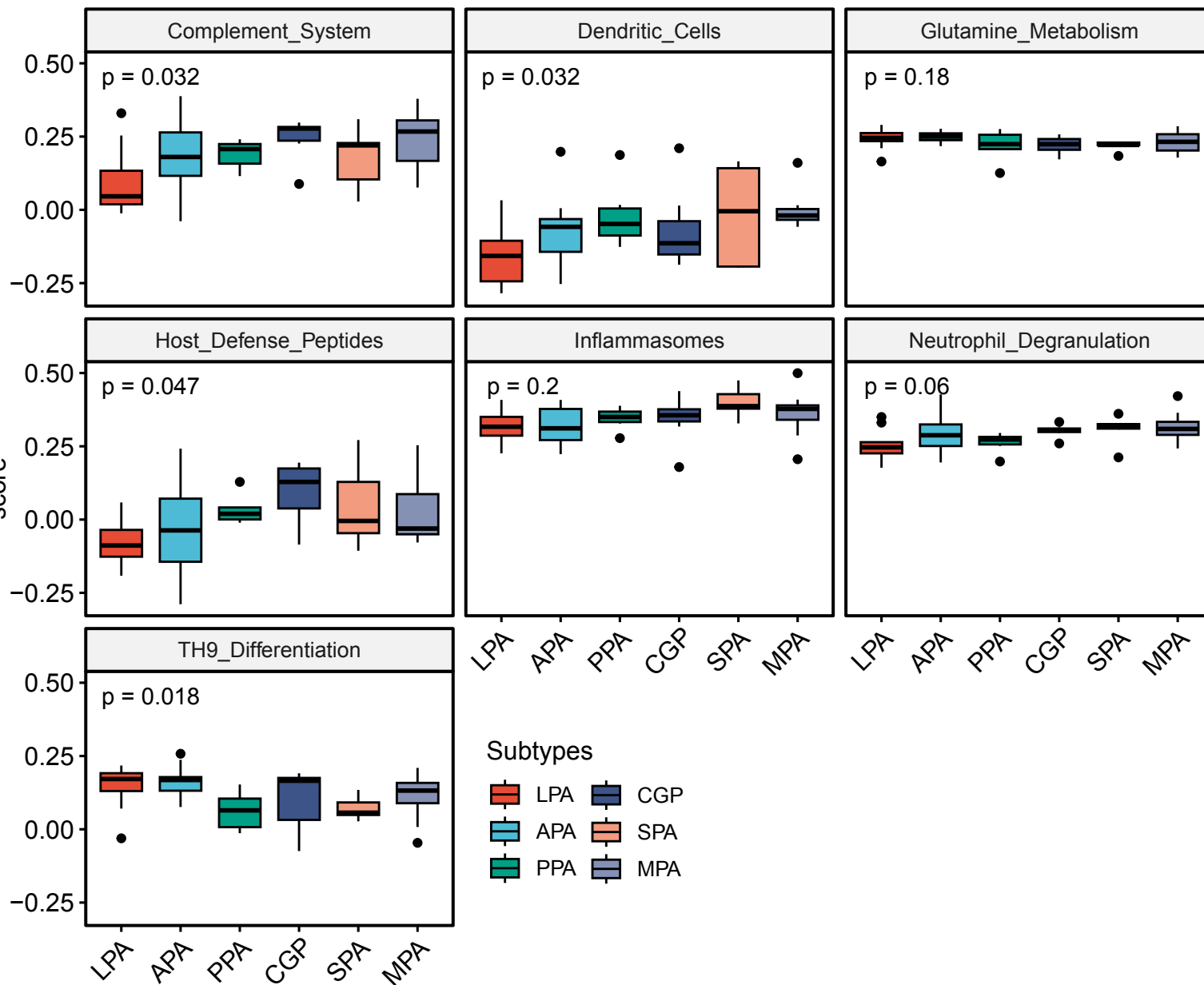

Supplement: Supplementary file 5 — Supporting Information [file CTM2-15-e70439-s013.pdf]

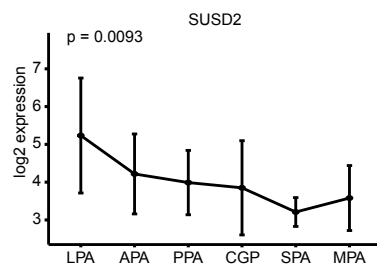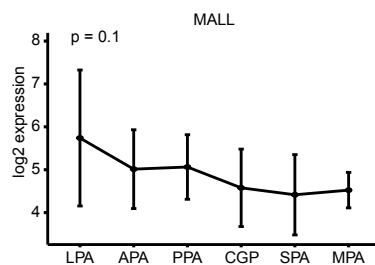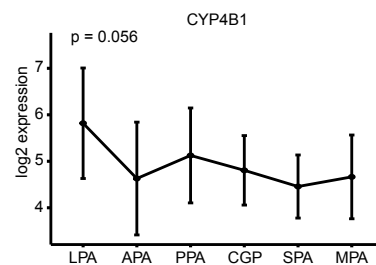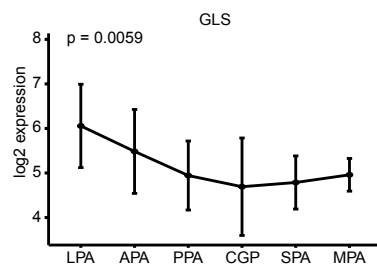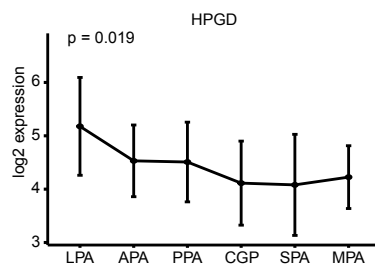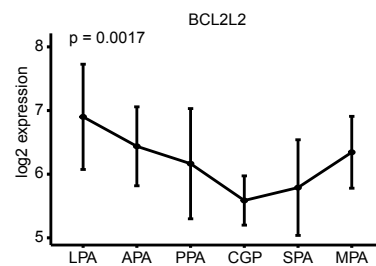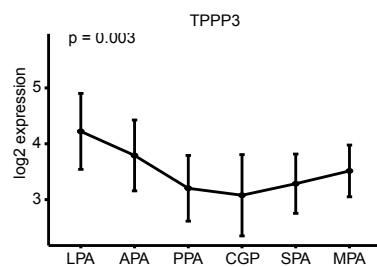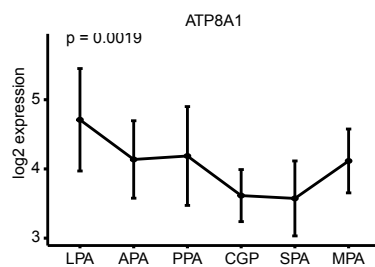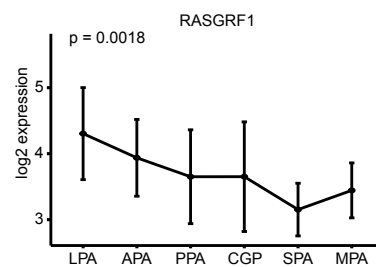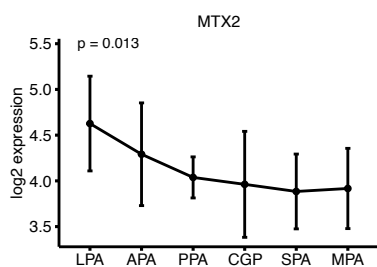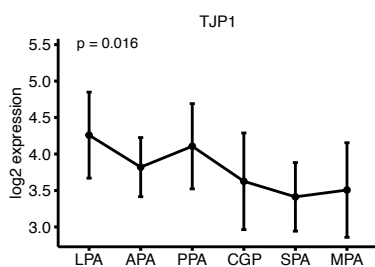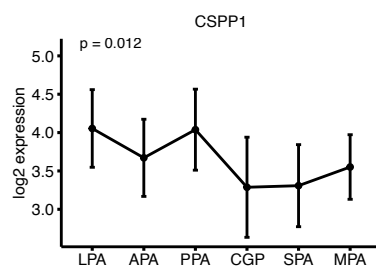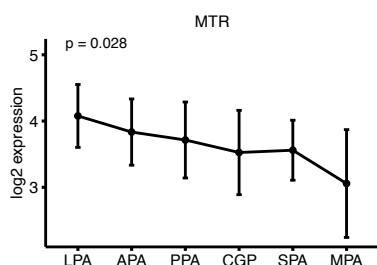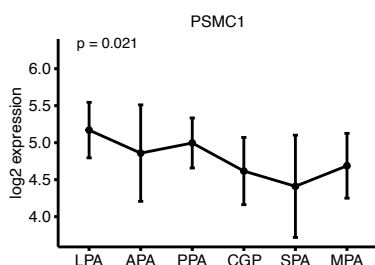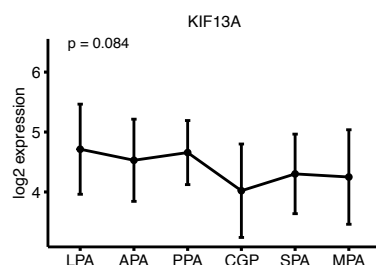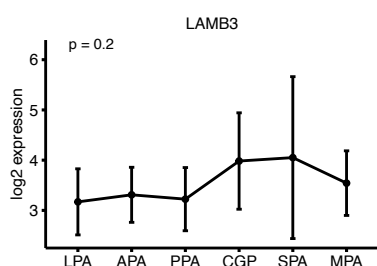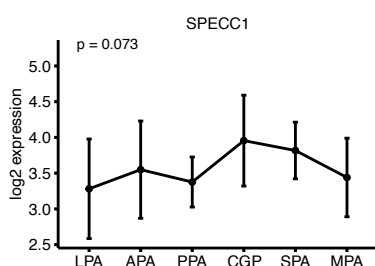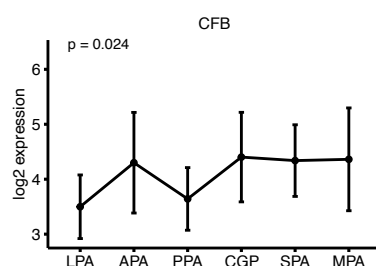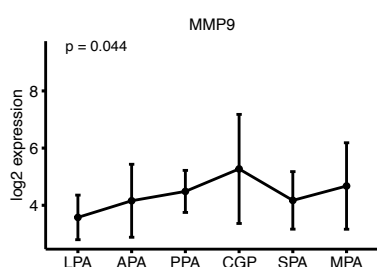

Supplement: Supplementary file 7 — Supporting Information [file CTM2-15-e70439-s001.pdf]

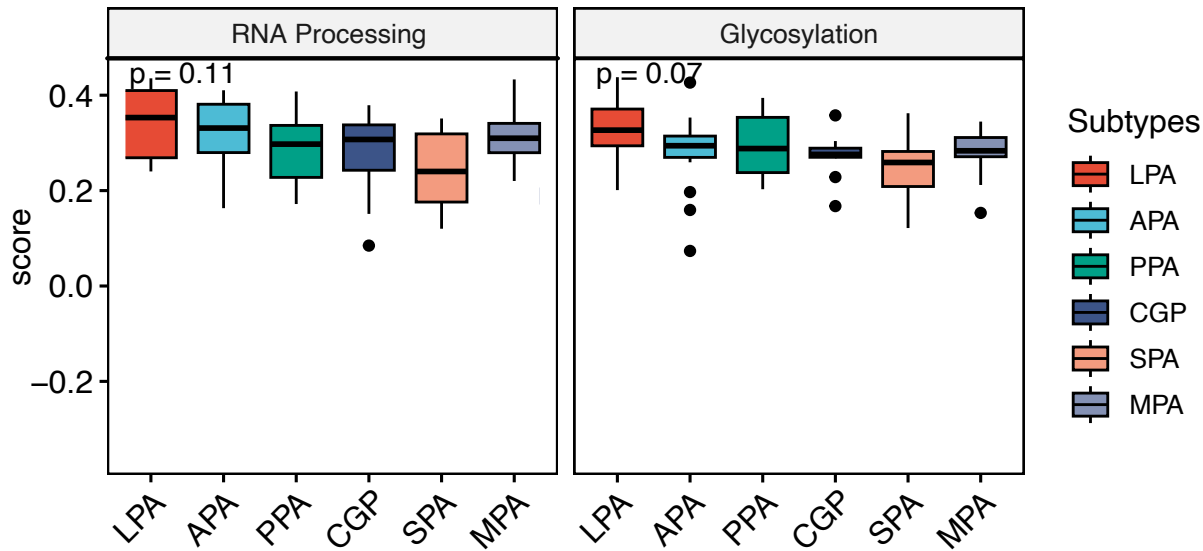

Supplement: Supplementary file 8 — Supporting Information [file CTM2-15-e70439-s009.pdf]

A

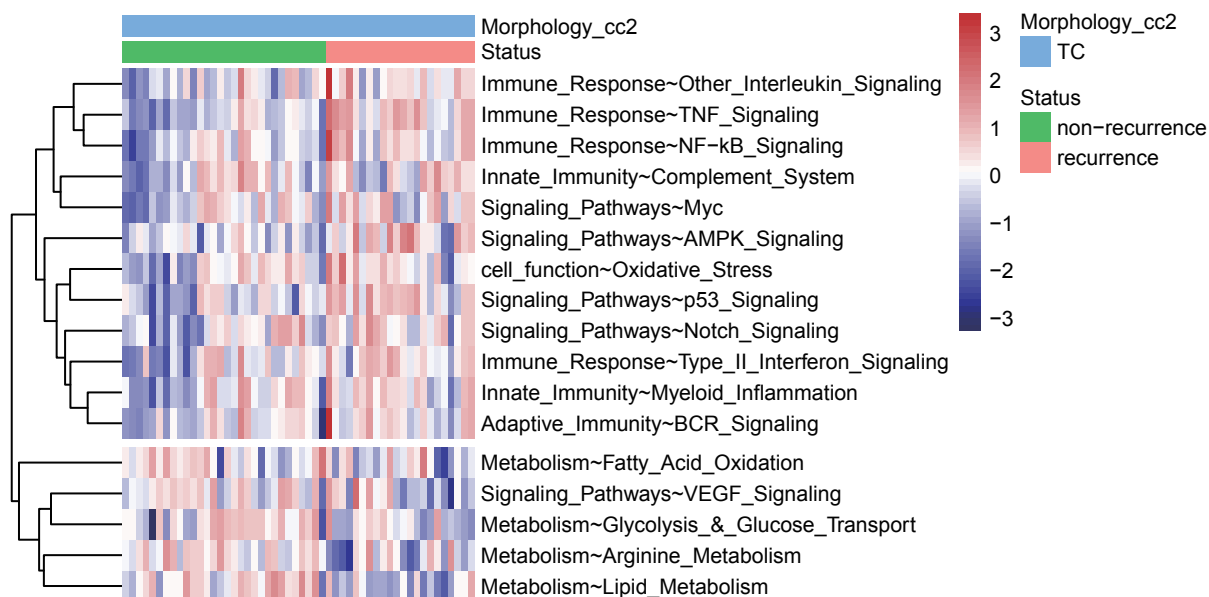

B

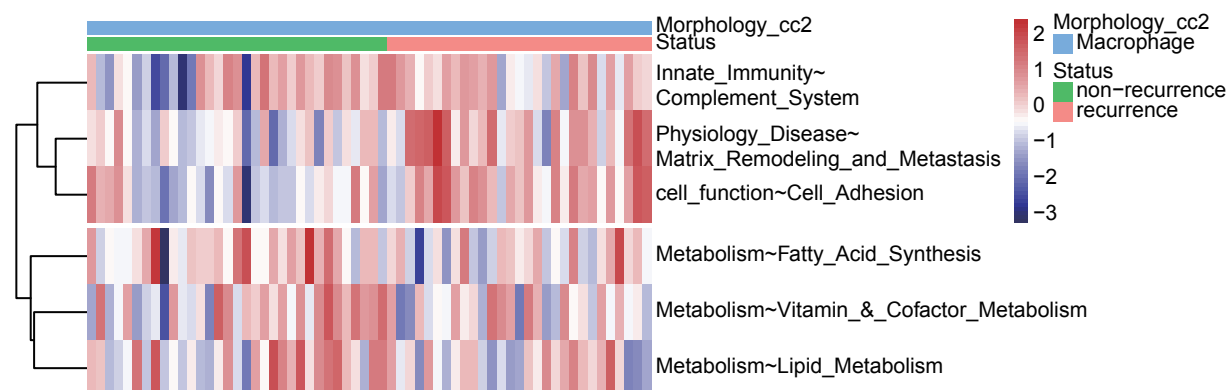

Supplement: Supplementary file 9 — Supporting Information [file CTM2-15-e70439-s015.pdf]

A

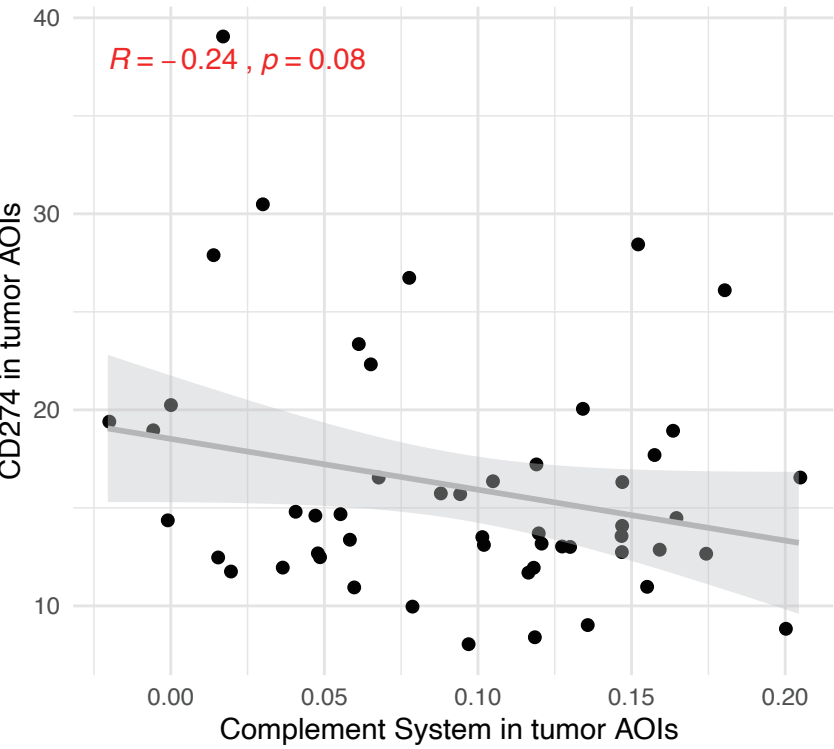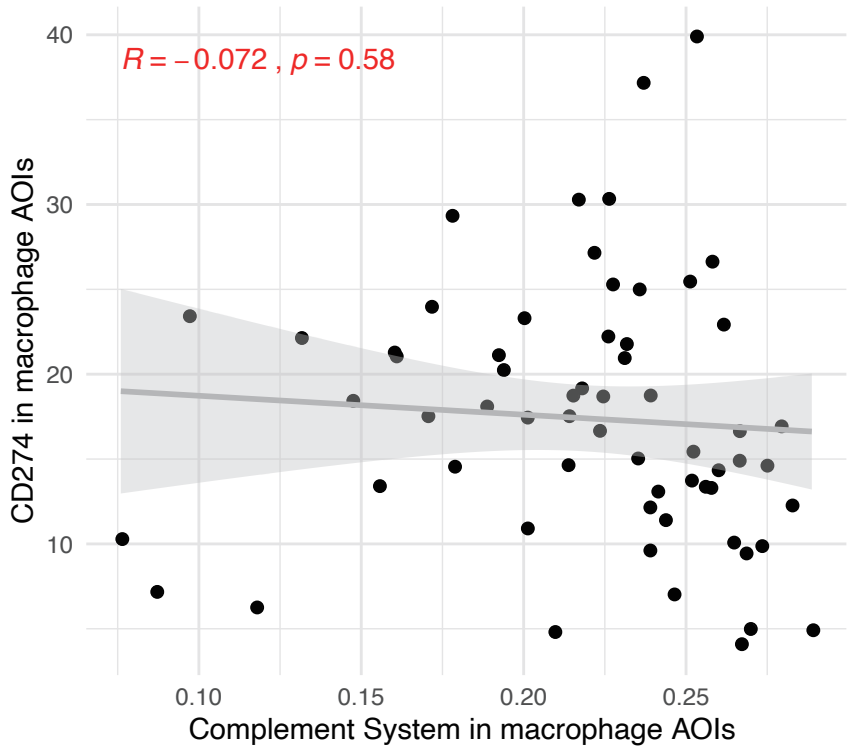

B

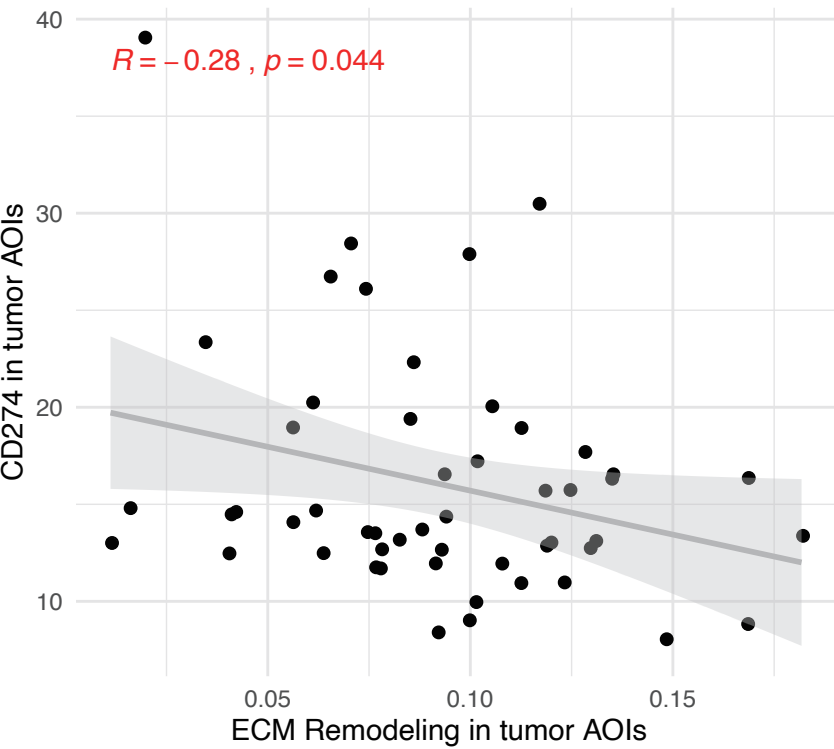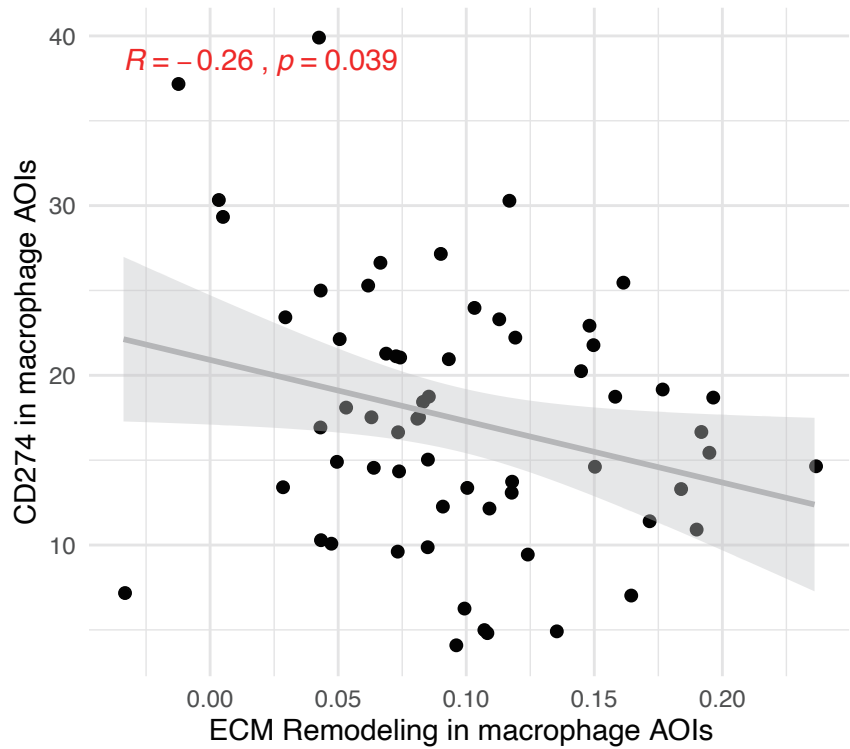

C

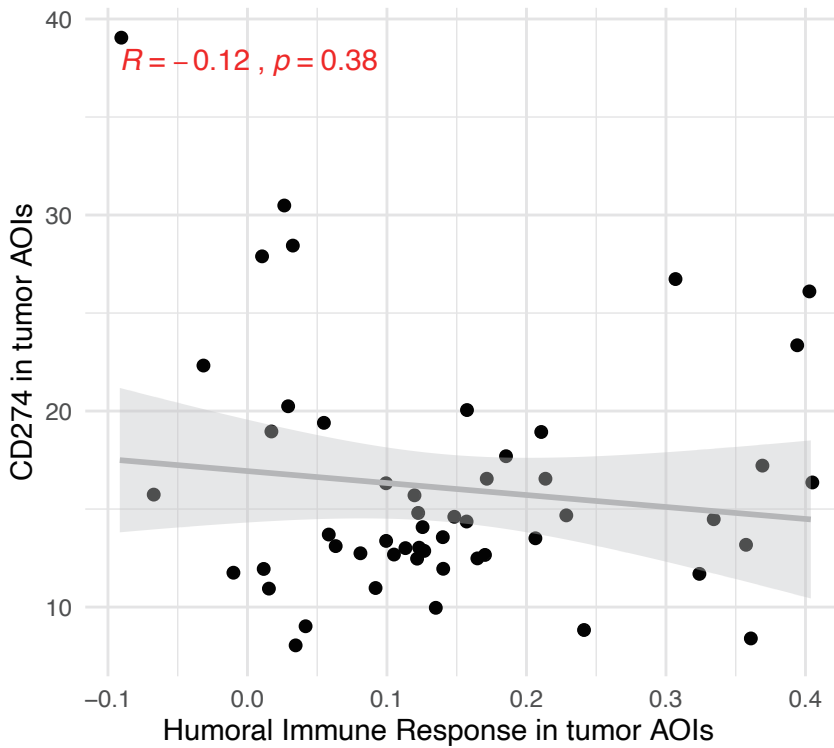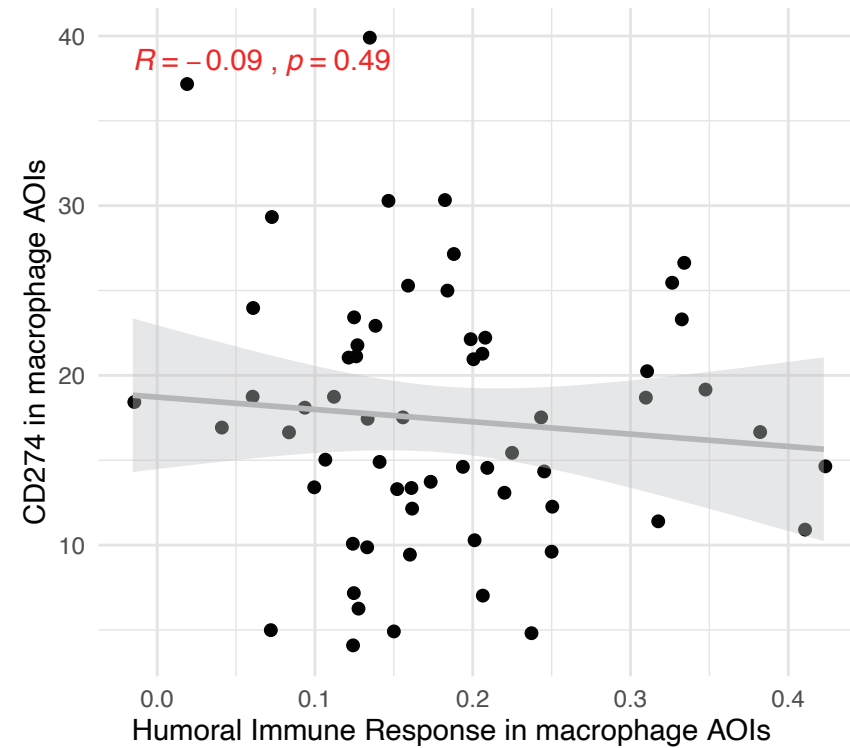

Supplement: Supplementary file 10 — Supporting Information [file CTM2-15-e70439-s014.pdf]

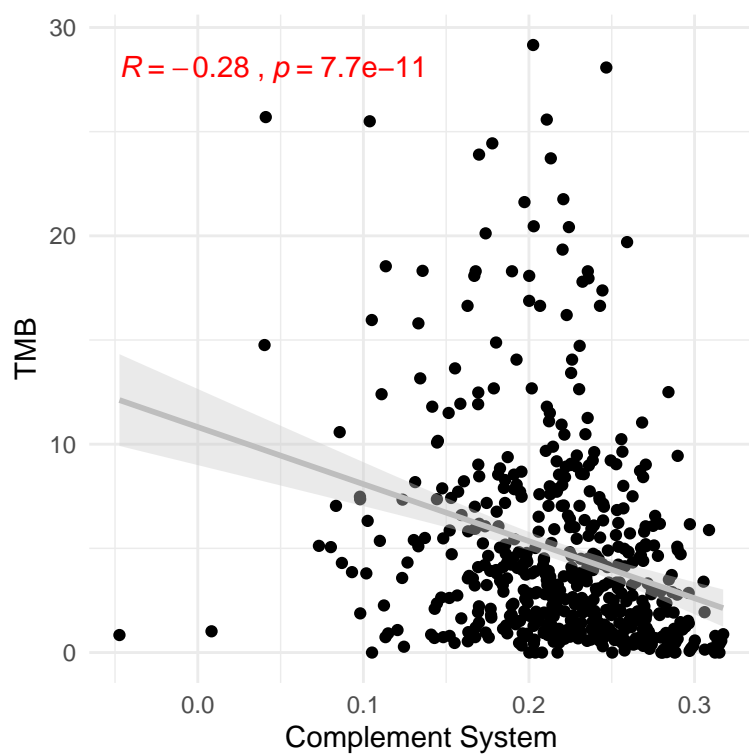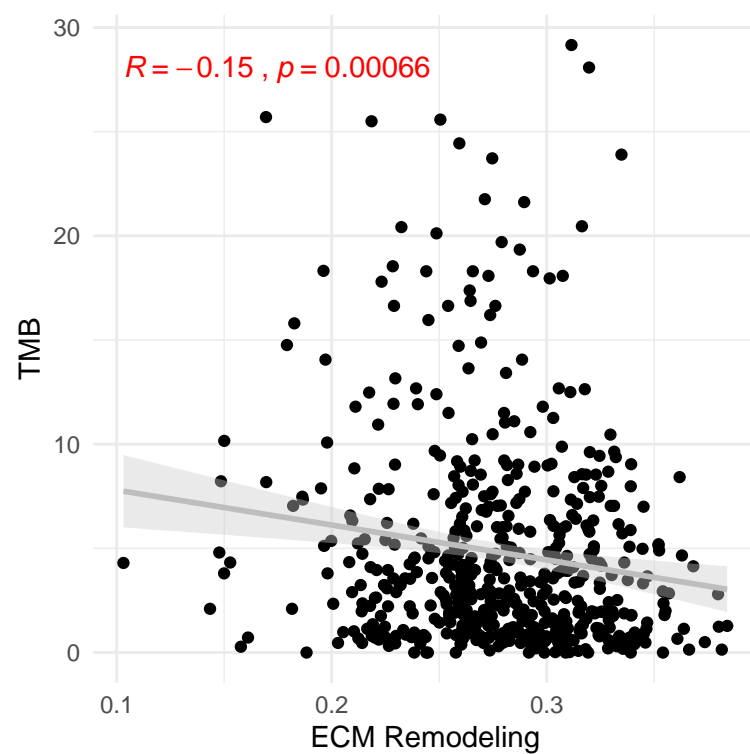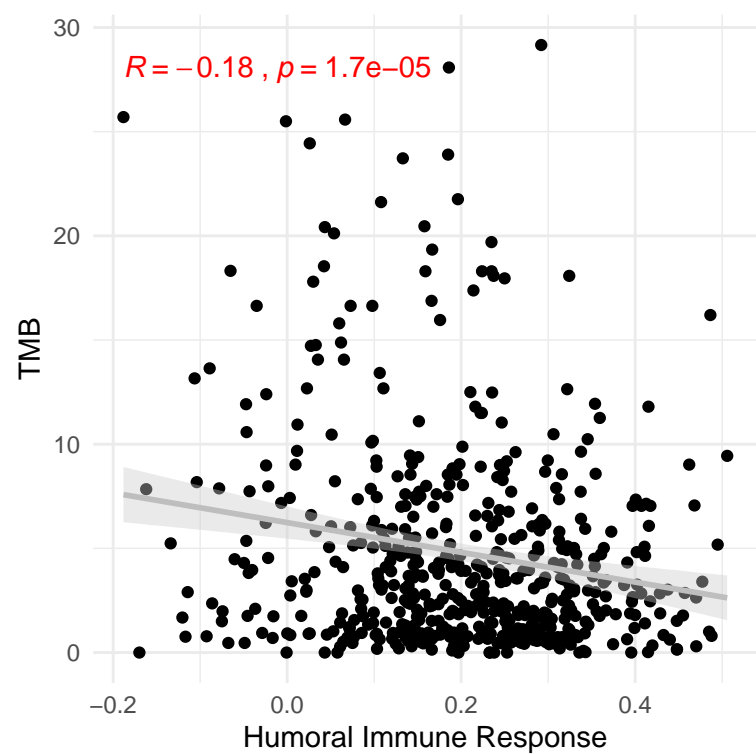

Supplement: Supplementary file 11 — Supporting Information [file CTM2-15-e70439-s005.pdf]

## SPP1

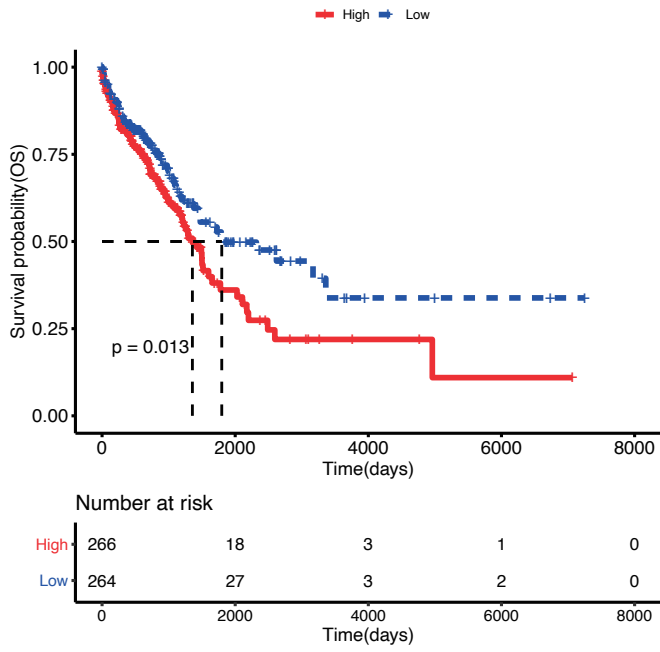

## MUC5B

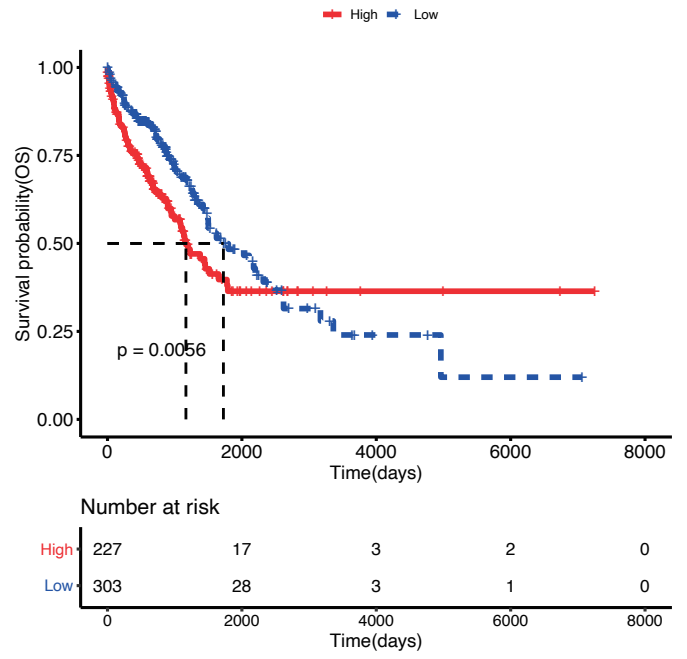

## CFB

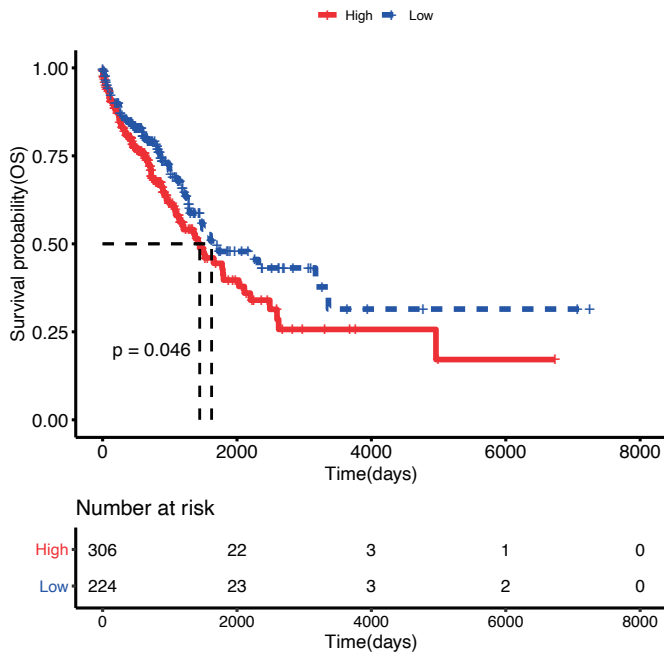

## CCL20

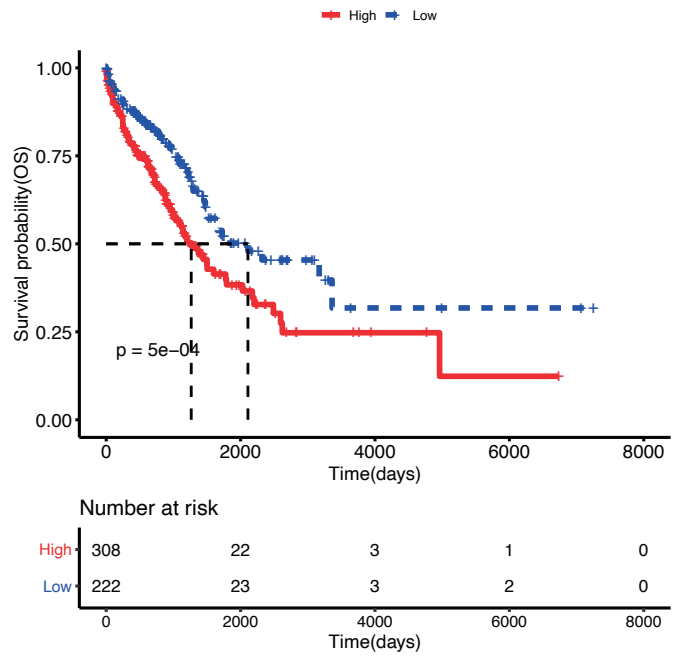

Supplement: Supplementary file 12 — Supporting Information [file CTM2-15-e70439-s011.pdf]
