# Supplementary material for: Spatial transcriptomic analysis across histological subtypes reveals molecular heterogeneity and prognostic markers in early‐stage lung adenocarcinoma
Source: Clin Transl Med. 2025 Aug 22;15(8):e70439. doi: 10.1002/ctm2.70439 (PMC12373976; doi:10.1002/ctm2.70439)
Supplement: Supplementary file 6 — Supporting Information [file CTM2-15-e70439-s012.pdf]

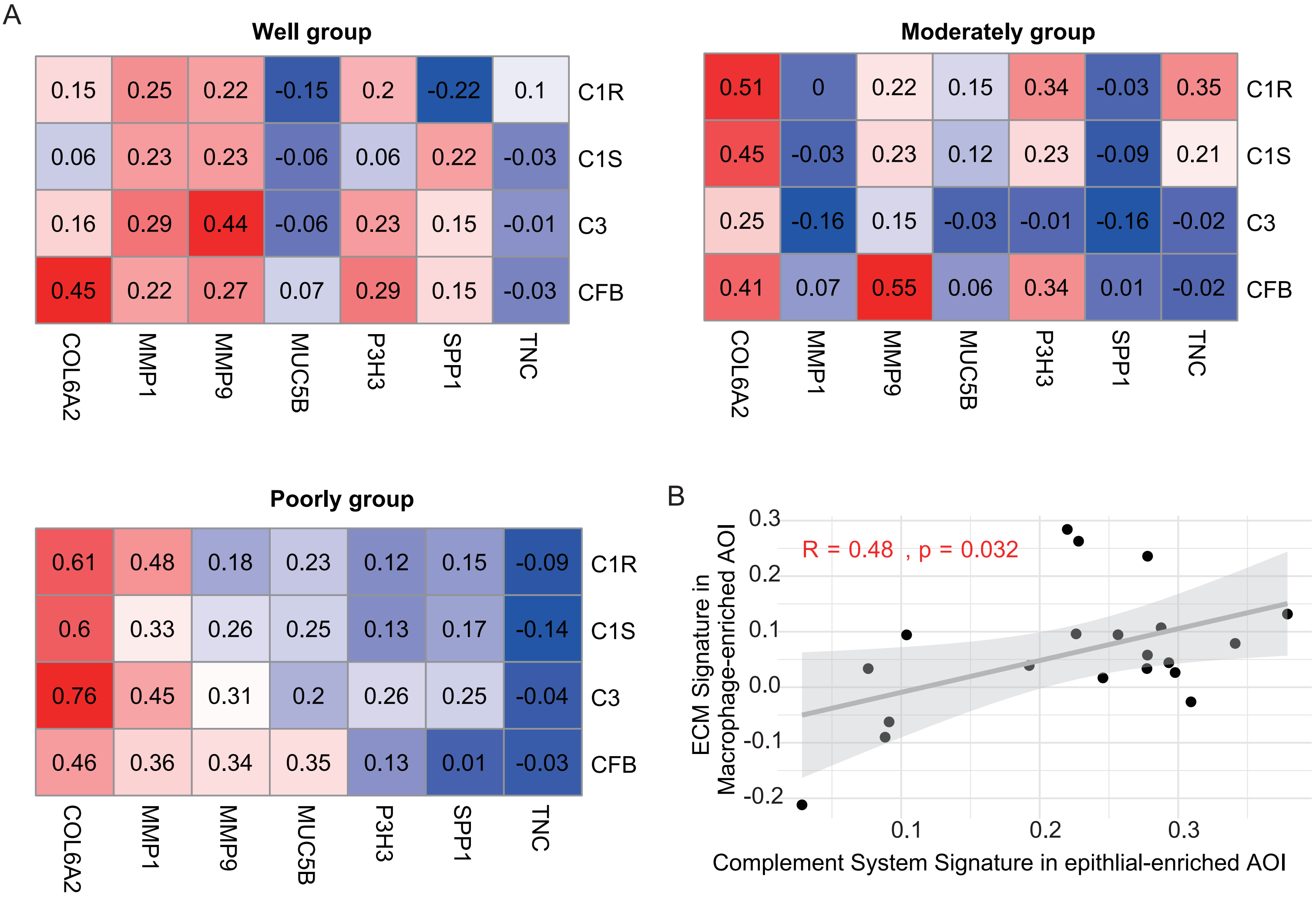
A
Well group
0.15 0.25 0.22 -0.15 0.2 -0.22 0.1 C1R
0.06 0.23 0.23 -0.06 0.06 0.22 -0.03 C1S
0.16 0.29 0.44 -0.06 0.23 0.15 -0.01 C3
0.45 0.22 0.27 0.07 0.29 0.15 -0.03 CFB
COL6A2 MMP1 MMP9 MUC5B P3H3 SPP1 TNC
Moderately group
0.51 0 0.22 0.15 0.34 -0.03 0.35 C1R
0.45 -0.03 0.23 0.12 0.23 -0.09 0.21 C1S
0.25 -0.16 0.15 -0.03 -0.01 -0.16 -0.02 C3
0.41 0.07 0.55 0.06 0.34 0.01 -0.02 CFB
COL6A2 MMP1 MMP9 MUC5B P3H3 SPP1 TNC
Poorly group
0.61 0.48 0.18 0.23 0.12 0.15 -0.09 C1R
0.6 0.33 0.26 0.25 0.13 0.17 -0.14 C1S
0.76 0.45 0.31 0.2 0.26 0.25 -0.04 C3
0.46 0.36 0.34 0.35 0.13 0.01 -0.03 CFB
COL6A2 MMP1 MMP9 MUC5B P3H3 SPP1 TNC
B
R = 0.48 , p = 0.032
ECM Signature in Macrophage-enriched AOI
Complement System Signature in epithlial-enriched AOI
0.3 0.2 0.1 0.0 -0.1 -0.2
0.1 0.2 0.3
