# Supplementary material for: Spatial transcriptomic analysis across histological subtypes reveals molecular heterogeneity and prognostic markers in early‐stage lung adenocarcinoma
Source: Clin Transl Med. 2025 Aug 22;15(8):e70439. doi: 10.1002/ctm2.70439 (PMC12373976; doi:10.1002/ctm2.70439)
Supplement: Supplementary file 13 — Supporting Information [file CTM2-15-e70439-s002.pdf]

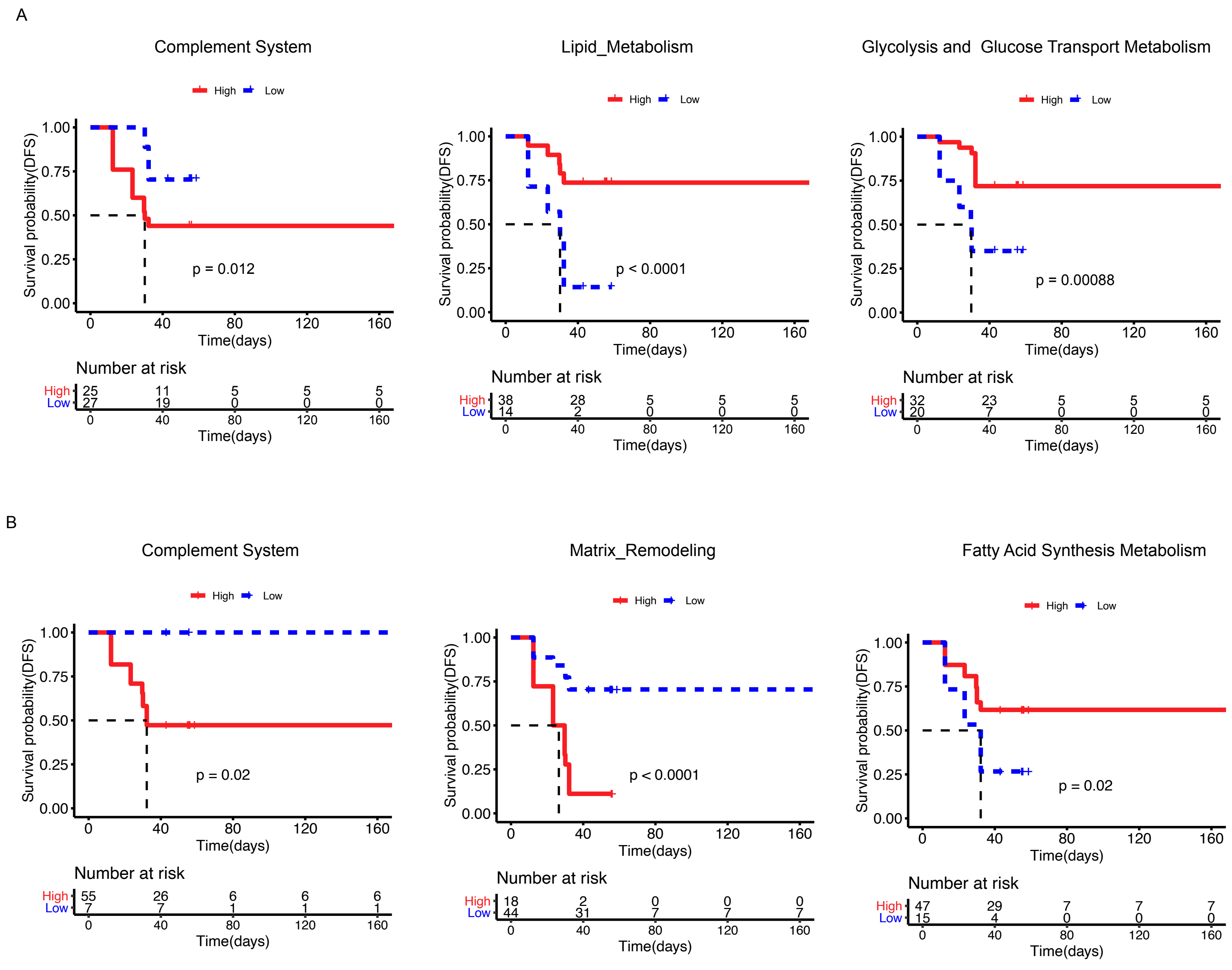
A
Complement System
High
Low
Survival probability(DFS)
1.00
0.75
0.50
0.25
0.00
p = 0.012
0
40
80
120
160
Time(days)
Number at risk
High 25 11 5 5 5
Low 27 19 0 0 0
Lipid_Metabolism
p < 0.0001
Number at risk
High 38 28 5 5 5
Low 14 2 0 0 0
Glycolysis and Glucose Transport Metabolism
p = 0.00088
Number at risk
High 32 23 5 5 5
Low 20 7 0 0 0
B
Complement System
p = 0.02
Number at risk
High 55 26 6 6 6
Low 7 7 1 1 1
Matrix_Remodeling
p < 0.0001
Number at risk
High 18 2 0 0 0
Low 44 31 7 7 7
Fatty Acid Synthesis Metabolism
p = 0.02
Number at risk
High 47 29 7 7 7
Low 15 4 0 0 0

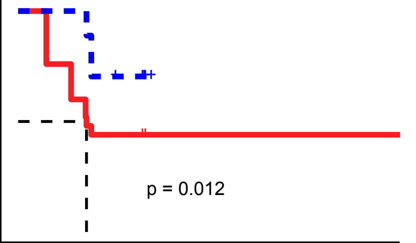
p = 0.012
